# Fluorinated Redox-Responsive Cross-Linked Poly(amidoamine) G2 as Smart Theranostic Dendrimers

**DOI:** 10.1021/acs.biomac.5c00914

**Published:** 2025-08-11

**Authors:** Carola Romani, Maria Cristina Bellucci, Maria Enrica Di Pietro, Paola Gagni, Mattia Sponchioni, Alessandro Volonterio

**Affiliations:** § Department of Chemistry, Materials and Chemical Engineering “Giulio Natta”, 18981 Politecnico di Milano, via Mancinelli 7, Milano 20131, Italy; ¥ Department of Food, Environmental, and Nutritional Sciences, 9304 Università degli Studi di Milano, via Celoria 2, Milano 20131, Italy; † Consiglio Nazionale delle Ricerche, Istituto di Scienze e Tecnologie Chimiche “Giulio Natta” (SCITEC), Via Mario Bianco 9, Milan 20131, Italy

## Abstract

This work presents a novel strategy for enhancing the gene delivery capabilities of low-generation poly­(amidoamine) (PAMAM) dendrimers by employing a fluorinated, redox-responsive cross-linker. The synthesized cross-linker, featuring both disulfide bonds and trifluoromethyl groups, facilitates efficient “click” Michael addition with PAMAM G2, producing cross-linked polymers with strong ^19^F nuclear magnetic resonance signals and favorable relaxation properties, enabling their use as ^19^F nuclear magnetic resonance tracers. The resulting fluorinated PAMAM G2 polymers also demonstrated excellent plasmid DNA complexation, glutathione-triggered degradation, and notably enhanced transfection efficiency, especially in cancerous HeLa cells notoriously associated with a reducing environment, while maintaining low cytotoxicity. The best-performing system, derived from PAMAM G2 reacted with the redox-responsive cross-linker in a 1:1 molar ratio, outperformed both unmodified PAMAM G2 and 25 kDa branched polyethylenimine, the current nonviral transfection gold standard. These findings highlight the potential of fluorinated bioreducible dendrimer networks as multifunctional, low-toxicity, and trackable platforms for efficient gene delivery and theranostic applications.

## Graphical abstract

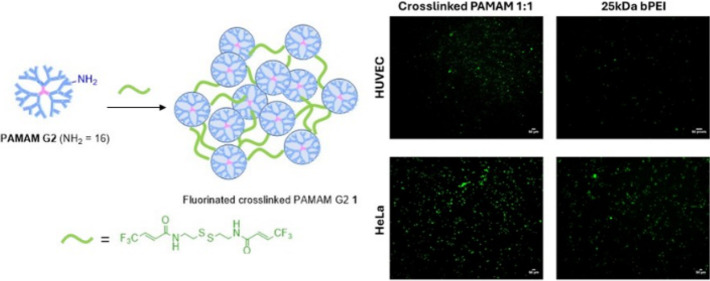

## Introduction

Since the pioneering work of Tomalia et al. in 1985,^1^ poly­(amidoamine) (PAMAM) dendrimers have become central figures in scientific research. These have been demonstrating particularly appealing in the realm of gene delivery, in the pursuit of alternatives to traditional viral carriers, whose utilization presents tough challenges, with immunogenicity being a primary concern.^2–6^ The inherent ability of PAMAMs to overcome the hurdles associated with delivering genetic material has positioned them as pivotal players in transfection research and therapeutic development. In this field, it was demonstrated that the transfection efficiency of PAMAM increases with higher generations, owing to their larger size, increased positive surface charge density, and complex branching structure. These properties facilitate the formation of stable complexes with nucleic acids, such as DNA or RNA, promoting an enhanced cellular uptake. A higher PAMAM generation also contributes to the dendrimer ability to protect nucleic acids from degradation and promote endosomal escape, critical steps for successful gene delivery.^7^ However, as the generation of PAMAM dendrimers increases, so does the potential for cytotoxic effects. Their properties may lead to interactions with cell membranes, potentially causing membrane disruption and cell death, revealing the necessity for active work to balance the improved transfection efficiency while minimizing cytotoxicity.^8^ Functionalization of the PAMAM dendrimers exploiting the reactivity of the outer primary amines has been for long time the leading strategy to achieve this goal.^9^ For instance, functionalization with tumor targeting ligand provided systems with enhanced transfection efficiency (TE), increased biocompatibility, and controlled release behavior.^10–12^ Along with the functionalization of high generation PAMAM dendrimers with moieties able to mitigate the cytotoxicity, there is an increasingly growing interest for the decoration of low-generation PAMAM dendrimers to increase their TE because of the lower cost, time saving, and ease of preparation.^13–18^

Regardless the strategy used, recent studies have highlighted the effectiveness of incorporating fluorocarbon chains into polymeric gene vectors encompassing PAMAM dendrimers.^19^ This strategic functionalization enhances membrane permeability, leading to improved cellular uptake and cytosolic delivery of genes along with lowering the cytotoxicity.^20^ Additionally, fluorination proves instrumental in increasing endosomal escape, preventing degradation of encapsulated biomolecules in endosomes and ensuring their release into the cytosol for therapeutic effects.^21^ Moreover, fluorinated polymers, thanks to their antifouling behavior, demonstrated enhanced serum tolerance by reducing interactions with serum proteins and extracellular matrix components.^22^ Beyond gene delivery applications, fluorinated polymers also exhibit promise as contrast agents in magnetic resonance imaging (MRI), offering their inherent safety profile coming from the lack of metallic components often associated with classic MRI contrast agents. The direct visualization of fluorine atoms provides excellent imaging resolution and higher contrast, facilitated by the absence of other fluorine atoms in the body and hence a minimal background signal.^23^

As an alternative to or combined with the functionalization strategy, the design and preparation of low-molecular-weight (MW) dendritic-based nanoconstructs have raised growing interest as backups to traditional high-MW polymers.^24,25^ In particular, the cross-linking of dendrimers with stimuli-responsive molecules can be utilized to enhance the cationic surface of low-MW polymers, resulting in more flexible polymeric structures, which are decomposed into smaller fragments by a specific stimulus into the target milieu. Among the different biological stimuli exploitable at this scope, variation in redox potential from the extracellular environment to the cytosol represents an appealing trigger for addressing therapeutics for tumor cells. In fact, their cytosol has been demonstrated to be rich in glutathione (GSH), making this environment particularly reductive. Common redox-responsive cross-linkers include cysteamine, cystine, disulfide-based dimethacrylates (DSDMAs), and *N*-succinimidyl-3-(2-pyridyldithio)­propionate (SPDP), all of which feature a central disulfide bond with (symmetrically) distributed functional groups at both ends.^26^ In this scenario, disulfide cross-linked low-generation PAMAM dendrimers G2 and G3, obtained using 3,3′-dithiodipropionic acid di­(*N*-succinimidyl ester) and 4,4’-dithiodibutirric acid, respectively, were described as efficient gene carriers able to condense pDNA into dendriplex, to efficiently transfect cancerogenic cells and to be degraded into the corresponding PAMAM monomers in the GSH-rich cytosol.^27,28^

These works inspired us to explore the synthesis of cross-linked low-generation PAMAM G2 with a fluorinated disulfide-containing cross-linker (Chart 1). To the best of our knowledge, this is the first time that the synthesis of a cross-linker bearing both a stimuli-responsive functional group and fluorinated moieties has been reported. The key features of the newly developed cross-linking agent derived from cysteamine rely on (1) the incorporation of two terminal vinylic trifluoromethyl (tfm) groups, which facilitate the “click” functionalization through Michael addition, a strategy we have developed and investigated for the functionalization of low-generation PAMAM dendrimers,^29–31^ enhancing the transfection efficiency of the nonviral vector and enabling key imaging capabilities, and (2) the presence of a central disulfide bond that degrades in a GSH-rich reducing environment, effectively mimicking the conditions found in tumor microenvironments.

**Chart 1:**
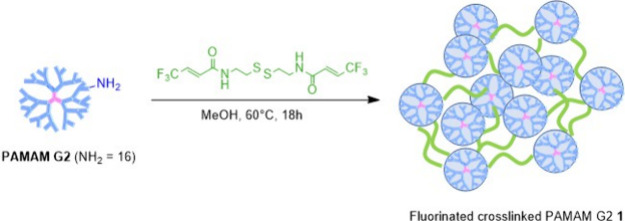
Synthesis of Fluorinated, Redox-Responsive Cross-Linked PAMAM G2 **1**

The developed fluorinated redox-responsive linker was used to build cross-linked PAMAM G2-based nanoclusters that can be degraded in a GSH-rich environment to low MW PAMAM G2 monomers, which we postulated could minimize the damage usually caused by high-MW cationic polymers. After a fine-tuning of the functionalization experimental settings and biophysical characterization, the most promising cross-linked PAMAM G2 polymer was evaluated in terms of TE and cytotoxicity in both noncancerogenic and cancerogenic cells, along with its properties as ^19^F MRI molecular tracers. The ultimate goal of this work is the synthesis of low cost, fluorinated, and redox-responsive low-generation PAMAM cross-linked dendrimers with high TE, low cytotoxicity, and suitable properties for implementation in ^19^F MRI applications.

## Experimental Section

### Materials

The PAMAM G2 dendrimer (ethylenediamine core, 16 and 64 surface groups), 25 kDa bPEI (*M*_w_ ∼25 kDa by LS, average *M*_n_ ∼10 kDa by GPC), and all other organic reactants, solvents, and culture reagents were purchased from Sigma-Aldrich (Milan, Italy) if not differently specified and used as received. The use of a cationic polymer based on PEI for transfection is covered by US Patent 6,013,240, European Patent 0,770,140, and foreign equivalents, for which Polyplus-transfection is the worldwide exclusive licensee. Spectra/Por dialysis bags (MWCO = 1 and 8 kDa) were from Spectrum Laboratories (Compton, CA, USA). The green fluorescent plasmid (Monster Green Fluorescent Protein phMGFP Vector) encoding for the modified green fluorescent protein was purchased from Promega (Milano, Italy). Cell viability was evaluated via MTT assays, and the HUVEC and HeLa cell lines were purchased from PromoCell (Heidelberg, Germany).

^1^H, ^13^C, and ^19^F NMR spectra were recorded on 400 MHz spectrometers. Chemical shifts are expressed in ppm (δ), using tetramethylsilane (TMS) as the internal standard for ^1^H and ^13^C nuclei (δH and δC = 0.00), while C_6_F_6_ was used as the external standard (δF −162.90) for 19F. ESI mass spectra were recorded by a Bruker Esquire 3000+ instrument equipped with an MS detector composed of an ESI ionization source and a single quadrupole mass-selective detector. Purification of the intermediates was performed by flash chromatography (FC) with Biotage Isolera one flash purification chromatography ISO–1SV Unit4 Pred Selekt.

### Synthesis of (2*E*,2′*E*)-*N*,*N*′-(Disulfanediylbis­(ethane-2,1-diyl))­bis­(4,4,4-trifluorobut-2-enamide) CBtfmA **2**

Cystamine dihydrochloride (200 mg, 1.3 mmol) was dissolved in DMF (13.2 mL) and stirred in the presence of DIPEA (0.45 mL, 2.6 mmol, 2 equiv) for 30 min. To this stirred solution were added solid trifluorocrotonic acid (367 mg, 2.6 mmol, 2 equiv), EDC x HCl (277 mg, 1.4 mmol, 1.1 equiv), and HOBt (195 mg, 1.4 mmol, 1.1 equiv). The solution was stirred at rt for 16 h. The solution was diluted with EtOAc and washed once with a 1 M aqueous solution of HCl, once with brine, once with a saturated aqueous solution of NaHCO_3_, and three times with brine. The organic phase was dried over Na_2_SO_4_, filtered, and the solvent evaporated under pressure affording 345 mg of **2** (1.13 mmol, 87% yield) as a white solid.

### CBtfmA **2**

^1^H NMR (CD_3_OD, 400 MHz) δ 6.63–6.61 (m, 2H), 3.53–3.50 (m, 2H), 2.78 (t, *J* = 6.4 Hz, 1H); ^19^F NMR (CD_3_OD, 376 MHz) δ −66.5 (d, *J* = 3.8 Hz); ^13^C NMR (CD_3_OD, 101 MHz) δ 165.0, 132.7 (q, *J* = 6.1 Hz), 128.4 (q, *J* = 35.3 Hz), 124.1 (q, *J* = 269.7 Hz), 39.8, 38.1; ESI *m*/*z* 397.1 [M+H, (100)]^+^; Anal. Calcd for C_12_H_14_F_6_N_2_O_2_S_2_: C, 36.36; H, 3.56; N, 7.07; found: C, 36.38; H, 3.56; N, 7.06.

### Synthesis of Fluorinated Cross-Linked PAMAM **1**: General Procedure

To a solution of CBtfmA **2** (*n* × 1.0 equiv, where *n* is the number of equiv with respect to the dendrimer) in MeOH (0.5 mL), a solution of PAMAM G2 (10 mg) in MeOH (0.5 mL) was added dropwise, and the resulting solution was stirred for 48 h at 60 °C. The solvent was evaporated, and the crude was purified via dialysis with a SpectraPor regenerated cellulose membrane (MWCO = 1 kDa) against deionized water for 2 days. The products were lyophilized, obtaining fluorinated cross-linked PAMAM as fluffy white solids. Yields of **1**(4-1), **1**(2-1), **1**(1-1), **1**(1-2), and **1**(1-4): 20% (2,0 mg), 35% (3,5 mg), 58% (5,8 mg), 34% (3,4%), 25% (2,5 mg).

### ^19^F NMR Experiments

#### ^1^H–^1^H COSY Experiment

The COSY spectrum was recorded using a data matrix of 1024 (*t*_2_) × 256 (*t*_1_) complex data points, with 80 transients per increment, 4 dummy scans, and a 2 s relaxation delay. Raw data were processed by applying cosine squared sine window functions in both dimensions.

#### ^19^F *T*_1_ and *T*_2_ NMR Measurements

High-resolution liquid-state NMR measurements were performed at 298 K at a concentration of 10 mg/600 μL without sample spinning with a Bruker NEO 500 console (11.74 T) equipped with a direct observe broadband including fluorine (BBFO) iProbe and a variable-temperature unit (^19^F resonance frequency of 470.59 MHz). The instrument was carefully tuned and shimmed, and the 90° pulses were calibrated. *T*_1_ and *T*_2_ relaxation measurements were performed with inversion recovery (IR) and Carr–Purcell–Meiboom–Gill (CPMG) pulse sequences, respectively. All relaxation measurements were carried out with relaxation delays at least five times the *T*_1_. The spin–lattice and spin–spin relaxation rates were measured using data matrices of 8192 (*t*_2_) × 16 (*t*_1_) complex data points over a spectral width of 20 ppm with a total of 32 transients per increment. The delay time τ ranged from 0.01 to 2 s in *T*_1_ experiments and from 0.008 to 0.016 s to 1.6–2 s in *T*_2_ experiments. The baselines of all arrayed *T*_1_ and *T*_2_ spectra were corrected prior to processing the data. Data were processed using an exponential filter in F_2_ dimension (with LB equal to 8 Hz), and integrals were used in calculating relaxation times. Relaxation times were computed from experimental raw data using the standard one-component fitting function in the Bruker *T*_1_/*T*_2_ relaxation module.

### DLS Analysis

Particle size distribution and ζ potential were assessed via dynamic light scattering (DLS). The analysis was performed on a Zetasizer Nano ZS (Malvern Instruments), comparing dendrimers obtained before and after complexation with the gene cargo. The analysis was performed with backscattering (173°) on samples prepared at the same concentrations as they were for transfections. The considered concentrations were reached exploiting 20 μL of 10 mg/mL aqueous dendrimer suspension added to 5 μL of water (nonviral vectors aqueous suspension) and 4 μL of 1 μg/μL DNA mixed with 1 μL of 100 mM NaOAc (gene suspension) separately prepared. Based on the desired w/w to achieve, a fraction of dendritic solution was mixed with 5 μL of the aqueous phase containing the genetic material, and the dendriplex suspension was left to complex for about 10 min at room temperature. Following a proper dilution in PBS to reach 0.6 μg of DNA dose, the dendriplex suspension was prepared to a final volume of 1.6 mL for a reliable analysis.

### Agarose Gel Retardation Assay

Dendrimers/DNA complexes at different w/w ratios were prepared considering 250 ng of pGFP. Complexes were then diluted in distilled water to a final volume of 20 and 5 μL of loading dye (Blue/Orange Loading Dye, 6× from Promega) and were added to the dendriplex suspension achieving a final volume of 25 μL for each sample to be loaded on 0.8% (w/v) agarose gel. In the first lane, the DNA ladder (BenchTop 1kb DNA Ladder, from Promega) was loaded, while free plasmid was loaded in the second lane. Electrophoresis running was done by maintaining the gel in Tris-acetate-EDTA buffer (1× TAE) at 100 V for 50 min. A plastic tray was used to take the gel and incubate it with the staining solution (Diamond Nucleic Acid Dye Promega diluted in 1× TAE buffer), following manufacturer’s instructions, for 20 min under gentle shaking, avoiding light sources. The gel analysis was performed under a UV Transilluminator BIO-RAD ChemiDoc XRS+ using proper filters.

### Transfection and Viability Protocols

Green fluorescent protein plasmid (monsterGFP plasmid from Promega) and HUVECs or HeLa cells were used for transfection efficiency and cytotoxicity evaluation for **1**(1-1). The transfection protocol involves the preparation of two separate solutions: 20 μL of 10 mg/mL aqueous dendrimer suspension plus 5 μL of water and 4 μL of 1 μg/μL DNA mixed with 1 μL of 100 mM NaOAc. A portion of dendritic suspension was mixed into 5 μL of aqueous phase containing the genetic material at a concentration calibrated to establish the desired w/w ratio, and the mixture was left to complex for about 10 min at room temperature. Then, following a proper dilution in PBS and then in a culture medium (Endothelial Cell Growth Medium MV from PromoCell) to reach a 0.6 μg DNA dose, 150 μL of medium/dendriplexes was added per well for 15,000 cells per well in a 96-well plate. For cytotoxicity tests without a plasmid cargo, the dendrimer suspension was mixed with 5 μL of PBS. Cell viability was evaluated via the MTT assay, which performed by replacing the culture medium with the same amount of 1× MTT+PBS/culture medium solution and incubating the cells for 4 h. After incubation, DMSO was added to dilute the MTT-formazan formed by metabolically active cells, and spectrophotometric measurement via a TECAN SPARK Multimode Microplate Reader of MTT-formazan at 570 nm was performed to quantify cell viability.

### Statistical Analysis

Four replicates were performed for each experimental condition, acquiring 10 images for each replicate with a CELENA S fluorescence microscope using PlanAchro 4× objective lens without prolonged light microscope exposure of culture cells. Student’s *t* test was used to determine statistical significance.

### Image Analysis for GFP Fluorescence and Percentage of Transfected Cells

For transfection efficiency studies, GFP fluorescence intensity measurement and the percentage of transfected cells were evaluated with ImageJ. The cell counting has been settled with the software analyzing for each image with specific tools Brightness & Contrast, Thresholding method, and Find Edges, to perform the cell count implemented with the Analyze Particle tool. The final percentage of transfected cells was obtained considering the ratio between the number of transfected cells and the total number of cells of the images acquired with a CELENA S with a GFP filter cube and in brightfield mode, respectively. The GFP fluorescence was evaluated as corrected total cell fluorescence (CTCF).

## Results and Discussion

### Synthesis of Fluorinated Cross-Linked PAMAM G2

With the aim of developing a redox-responsive cross-linker, which could be broadly exploited for the synthesis of bioreducible polymers with theranostic properties, i.e., able to act simultaneously as a therapeutic and diagnostic tool, we envisaged installing two trifluoromethyl groups into *N*′-bis­(acryloyl)­cystamine (CBA). This is, in fact, one of the most exploited redox-sensitive cross-linker for the formation of biodegradable, stimuli-responsive polymer networks, particularly in biomedical and drug delivery applications.^26^ Accordingly, *N*′-bis­(trifluoromethylacryloyl)­cystamine (CBtfmA) **2** was prepared in high yields starting from commercially available cystamine dihydrochloride by a neutralization and coupling reaction with 4,4,4-trifluorocrotonic acid (Scheme 1). The detailed proton nuclear magnetic resonance (^1^H NMR) characterization of **2**, confirming the success of the synthesis, is reported in the Supporting Information. It is worth noting that the presence of a highly electronegative tfm group in the β-position of an α,β-unsaturated carbon–carbon double bond renders the later less electron-rich, thus more reactive toward nucleophile, facilitating the “click” functionalization through Michael addition.^32–34^

**Scheme 1:**
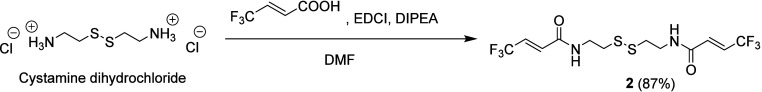
Synthesis of Fluorinated Cystamine-Derived Michael Acceptor CBtfmA **2**

After having obtained the redox-responsive cross-linker, we focused on its employment to reticulate PAMAM G2 dendrimers. To fine-tune the cross-linking experimental conditions, we first focused on the most appropriate temperature conditions between room temperature, 40 °C, and 60 °C working with PAMAM G2 dendrimer and CBtfmA **2** in an equimolar ratio ([PAMAM G2]/[CBtfmA **2**] = 1). The reaction was run in methanol at the given temperatures, and the evolution of the reaction was monitored by ^1^H and ^19^F NMR taking aliquots of 100 μL after 5, 9, 24, and 48 h and observing the ratio between the signals belonging to the starting materials versus those belonging to the final cross-linked polymer. As evidenced in Figures 1 and 2, the cross-linking did not occur at rt. Indeed, both the ^1^H and ^19^F NMR spectra recorded after 48 h (Figures 1A and 2A) showed the presence of the signal of the starting materials only. In particular, in the fluorine spectrum (Figure 2A), only the doublet belonging to the two equivalent tfm groups of linker CBtfmA **2** can be observed. After raising the temperature and observing especially the ^19^F spectra recorded, it is evident that the cross-linking occurs between the 24 and 48 h (Figure 2B,C). More in detail, the 48 h fluorine spectrum of the reaction performed at 40 °C clearly showed the presence of three peaks: one sharp peak and one broad peak resonating around −65.0 ppm belonging to the tfm groups of CBtfmA **2** and probably to a terminal vinylic tfm group of the cross-linker that has reacted at only one end and another broad peak at more negative ppm (around −76.5 ppm) belonging the tfm groups of the cross-linked PAMAM G2 **1**. The difference in chemical shifts of the fluorine signals is attributed to the tfm groups being attached to sp^3^ carbons in the cross-linked polymer, rather than to sp^2^ carbons as in diacrylate **2**. This confirms that the reaction is occurring successfully. Moreover, also in the ^1^H NMR spectra (Figure 1B), peaks related to cross-linked PAMAM G2 dendrimers **1** (in the blue circle) start to appear after 48 h. Indeed, these signals belong to the hydrogen in the α-position with respect to the tfm group and the methylene protons in the α-position to the nitrogen of the amidic functional group of the linker (see below). All these data suggest that the cross-linking does occur at 40 °C even if quite slowly. Finally, when the cross-linking reaction was run at 60 °C, looking at the ^19^F NMR spectrum (Figure 2C), it is evident that PAMAM G2 and diacrylate **2** underwent cross-linking successfully. In particular, the 48 h spectrum showed the presence of only traces of unreacted CBtfmA **2** along with the signal belonging to the cross-linked polymer **1**, which is much more intense, indicating that the reaction at 60 °C occurred with higher yield. It is worth noting that after 48 h, there is no evidence of the broad peak at −65.0 ppm that we observed at lower temperatures, indicating the absence of carbon–carbon double bonds bearing the tfm group in the final cross-linked PAMAM G2 dendrimer **1**. This could be seen also in the ^1^H NMR spectrum (Figure 1C), where the peaks belonging to the cross-linked PAMAM G2 dendrimers **1** (encircled in blue) are more intense, particularly in the spectrum recorded after 48 h (see also Figure S1 in the Supporting Information, which shows the disappearance of the olefinic signal of the cross-linker). Given the success of the cross-linking at 60 °C, the particle size distribution of the suspension obtained during this experiment was monitored via dynamic light scattering (DLS) after 5, 9, 24, and 48 h (Figure S2 the Supporting Information). Initially, there is a significant increase in particle size, reaching its peak after 24 h incubation, followed by a subsequent stabilization around 190 nm, suggesting a progressive enlargement of the structure over time because of cross-linking. All these observations evidenced that the best experimental conditions are 60 °C and 48 h.

**Figure 1:**
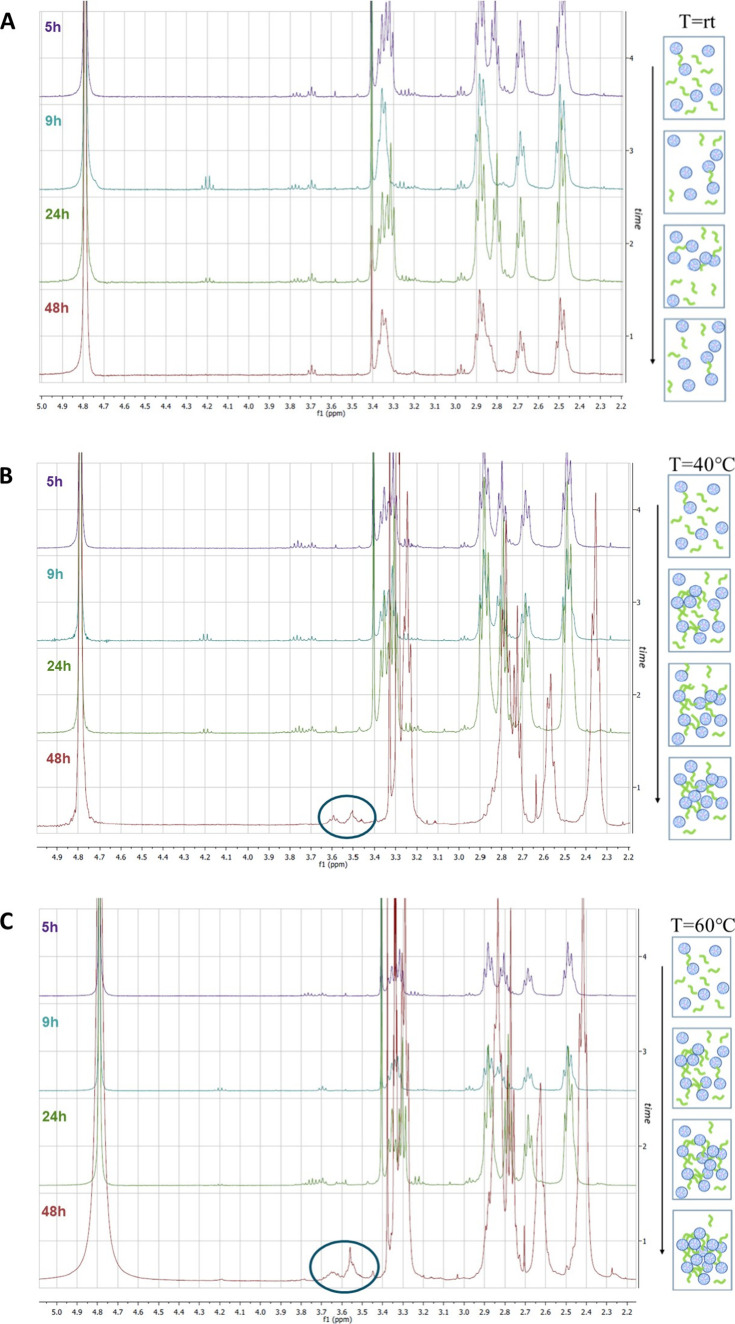
Kinetic study by ^1^H NMR of cross-linked structure **1** (molar ratio 1:1) at (A) rt, (B) 40 °C, and (C) 60 °C.

**Figure 2:**
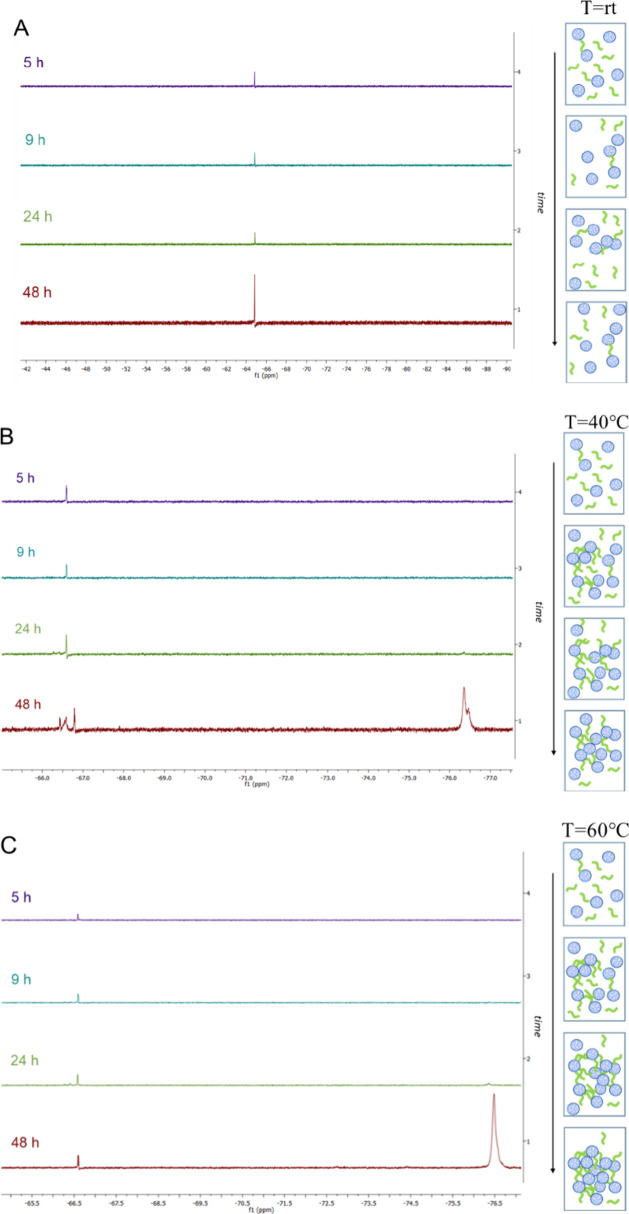
Kinetic study by ^19^F NMR of cross-linked structure **1** (molar ratio 1:1) at (A) rt, (B) 40 °C, and (C) 60 °C.

After the determination of optimal temperature and reaction time, various molar ratios (PAMAM G2/diacrylate **2**), including 4:1, 2:1, 1:1, 1:2, and 1:4, were tested and the corresponding polymers named **1**(4-1), **1**(2-1), **1**(1-1), **1**(1-2), and **1**(1-4) respectively. The reaction mixtures were stirred for 48 h at 60 °C in methanol, and the fluorinated cross-linked PAMAM obtained was characterized by ^1^H NMR and ^19^F NMR. As evidenced by the stacked ^19^F NMR spectra shown in Figure 3C, the reaction performed with a large excess of PAMAM G2 dendrimer did not produce a cross-linked polymer **1**(4-1) with suitable MW. Indeed, after lyophilization of the mixture (see experimental procedures), the few milligrams recovered did not show any detectable ^19^F signal. On the contrary, we did recover cross-linked fluorinated polymers **1** by working with all the other molar ratios, even if polymers **1**(2-1), **1**(1-2), and overall **1**(1-4) were obtained in low yields (see the experimental procedures section).

**Figure 3:**
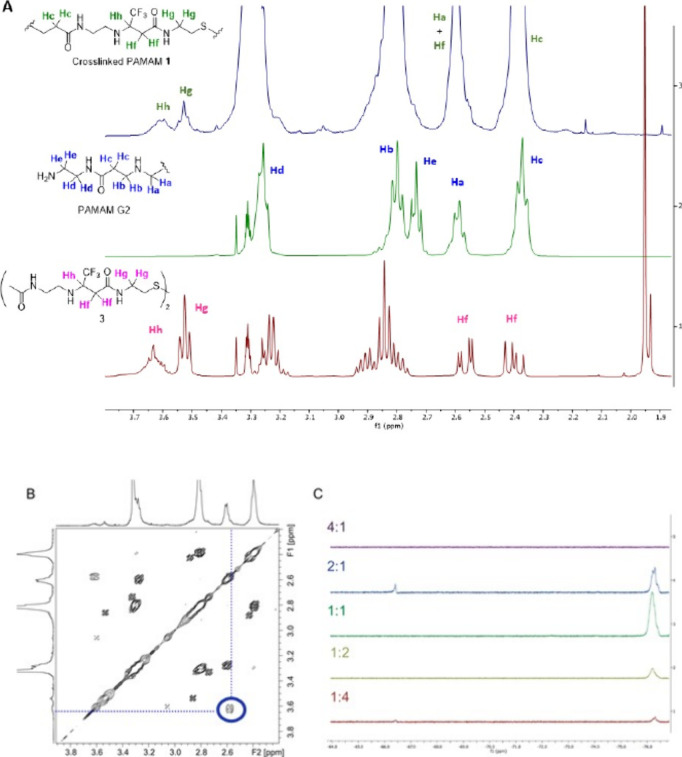
(A) ^1^H NMR spectra recorded in CD_3_OD of PAMAM G2 (green spectrum), model compound **3** (violet spectrum), and fluorinated cross-linked PAMAM **1**(1-1) (blue spectrum); (B) selected region of ^1^H–^1^H COSY NMR spectrum of fluorinated cross-linked PAMAM **1**(1-1); (C) ^19^F NMR spectra recorded for the cross-linked structures at different PAMAM/diacrylate molar ratios.

With the aim of using the resulting fluorinated polymer **1** as the imaging agent, the cross-linked polymer **1**(1-1) stands out as the most promising, marked by the presence of the most intense and well-defined fluorine signal peak.

The ^1^H NMR analysis facilitated the determination of the cross-linking ratio as the proportion of fluorinated diacrylate molecules forming covalent bonds with the outer primary amines of PAMAM G2 dendrimer. In Figure 3A, the ^1^H NMR spectra of cross-linked fluorinated PAMAM G2 **1**(1-1) (blue spectrum), undecorated PAMAM G2 (green spectrum), and model compound **3** was prepared by addition of two equivalents of *N*-(2-aminoethyl)­acetamide to **2** (see the Supporting Information, red spectrum), all recorded in CD_3_OD, are represented as an example. The two peaks resonating between 3.5 and 3.7 ppm in the spectrum of **1**(1-1), which are absent in the spectrum of the undecorated PAMAM dendrimer but clearly visible in the spectrum of model compound **3**, belong to the protons H_g_ and H_h_, namely, the two hydrogens of the methylene group in the α-position to the amidic nitrogen of the linker and the hydrogen vicinal to the tfm group, respectively. These could be integrated versus the broad peak resonating around 2.4 ppm belonging to the methylene protons in the α-position of the carbonyl groups in the PAMAM dendrimer (protons H_c_ which are 56 for PAMAM G2) to quantify the ratio **2**/PAMAM in the final cross-linked polymer **1**, a strategy already exploited in former works.^29–31^ However, the ^1^H NMR spectrum of model compound **3** showed two peaks resonating between 2.6 and 2.3 ppm belonging to the two diastereotopic methylene protons in the α-position to the carbonyl compounds, which form after the Michael addition (hydrogens H_f_). These signals could interfere with the integration if the corresponding H_f_ protons of the polymer **1** resonated at 2.4 ppm, namely, below the signal taken as reference for the PAMAM dendrimer. To assess that, we performed ^1^H–^1^H correlation spectroscopy (COSY) on the cross-linked polymer **1**(1-1). This clearly showed that proton H_h_ had a single cross-peak out of the diagonal (blue circle, Figure 3B) with the signal at 2.6 where the two H_f_ protons of the polymer resonate. This result confirms that the broad peak of **1**(1-1) at 2.4 ppm exclusively pertains to protons near the carbonyl group of PAMAM G2 dendrimers, eliminating ambiguity and solidifying its selection as the reference peak for subsequent analyses. In the ^1^H NMR spectra of the cross-linked PAMAM G2 **1**, the area under the signals were determined with reference to the integral of the peak generated by the hydrogens H_b_ of the PAMAM G2 moiety (56 protons, see spectra in the Supporting Information). The ratio between the integrated area of hydrogens H_h_ and H_g_ belonging to half of the fluorinated linker (C) and the number of these hydrogens, i.e., 3, gave the number of diacrylate molecules bonded to the PAMAM G2 dendrimer (Figure 4).

**Figure 4:**
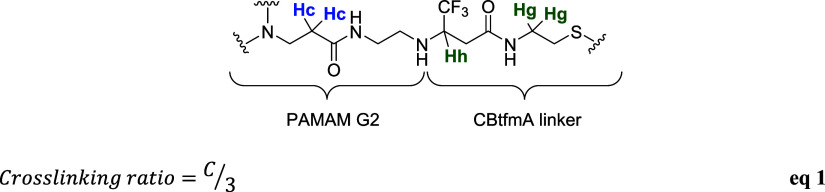
Hydrogens of cross-linked polymer **1** taken in consideration to compute the cross-linking ratio and the equation used (eq 1).

It is worth noting that the occurrence of possible intramolecular cyclization could affect the computation of the cross-linking ratio. However, the high concentration of PAMAM G2 in the reaction medium along with the presence of a large excess of amino functional groups should favor the intermolecular cross-linking process, minimizing the intramolecular cyclization.

The chemical characterization, comprising the chemical shift of the ^19^F NMR signals, and the calculated cross-linked ratio of all the dendrimers synthesized are summarized in Table 1.

**Table 1:**
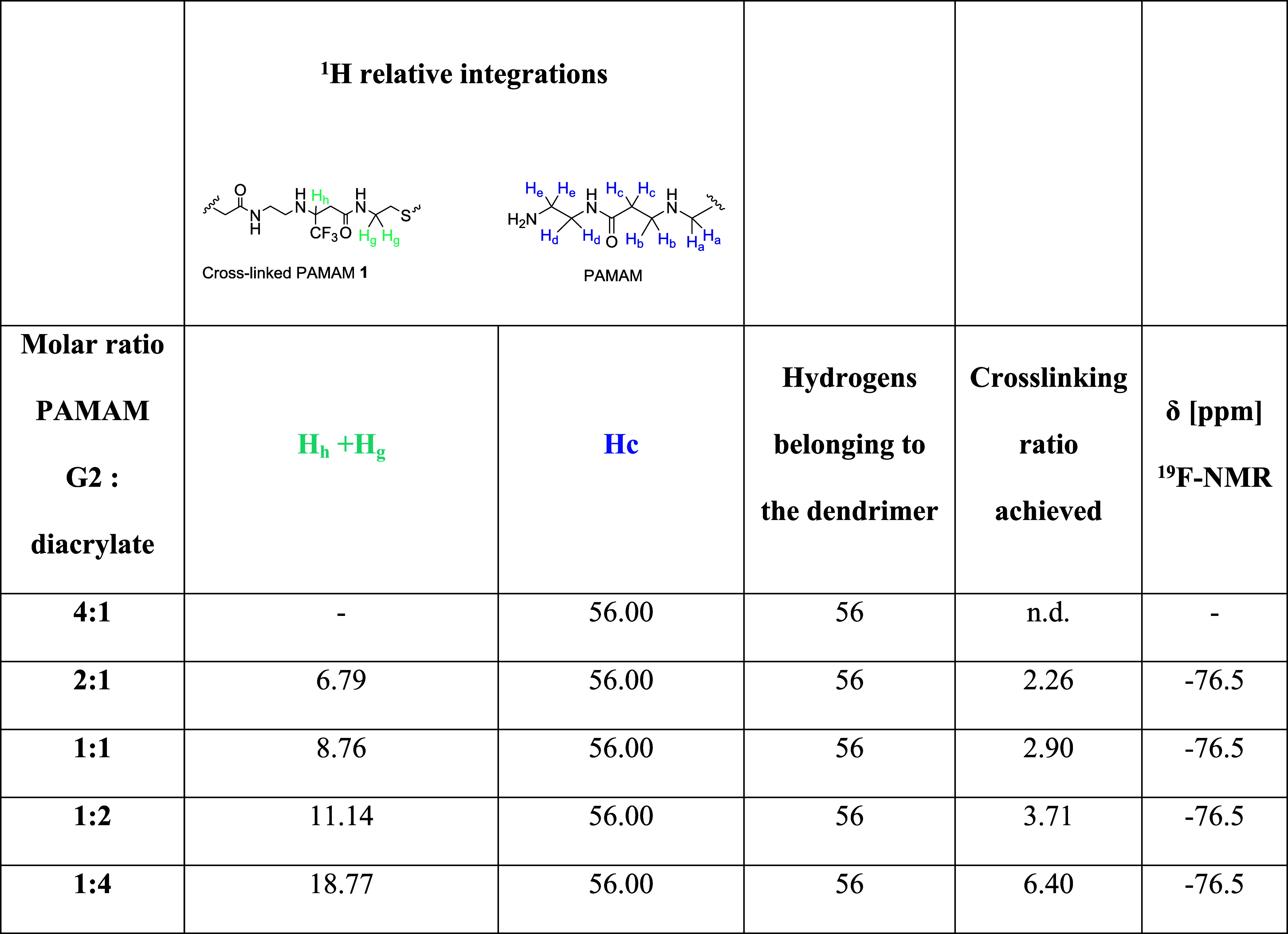
Chemical Characterization of Fluorinated Cross-Linked PAMAM G2 at Different Diacrylate:PAMAM Ratios

### Analysis of the ^19^F NMR Spectra of the Cross-Linked Polymer **1**

Over the past two decades, fluorine MRI has been extensively explored as a promising alternative to the hydrogen-based MRI commonly used in clinical practice.^35^ In this context, the development of fluorinated polymer tracers (FPTs) has witnessed an increasing interest due to several advantages over the most widely studied small-molecule perfluorocarbons, as their polymer structure, size, and architecture can be precisely engineered to impart specific physicochemical and biological properties.^36^ Notably, fluorinated stimuli-responsive polymers can change their physicochemical properties in response to specific stimuli, and incorporating noninvasive ^19^F labels enables real-time tracking and monitoring of environmental changes via alterations in ^19^F MRI signals.^37^ Since the obtained fluorinated cross-linked PAMAM G2 **1** showed a single, intense, and quite sharp ^19^F NMR peak, we sought to study more in detail the potentiality for a possible application in ^19^F MRI. Accordingly, spin–lattice *T*_1_ and spin–spin *T*_2_ relaxation times of the most promising candidate **1**(1-1) were measured using the standard inversion recovery (IR) and Carr–Purcell–Meiboom–Gill (CPMG) pulse sequences. Values of 296 and 136 ms were obtained for *T*_1_ and *T*_2_ relaxation times at 11.74 T, respectively, which are in line with previous studies on functionalized PAMAM G2 dendrimers,^31^ and particularly attracting in the current portfolio of (per)­fluorinated compounds.^38–40^ The favorable short longitudinal *T*_1_ time and acceptably long transverse *T*_2_ time make the cross-linked PAMAM **1**(1-1) suitable for an application as an ^19^F NMR contrast agent.

### Biophysical Characterization of Plasmid/Fluorinated Cross-Linked PAMAM G2 Complexes

Size analysis and complexation studies with a plasmid DNA encoding the green fluorescent protein (GFP) were performed using DLS and agarose gel retardation assay, respectively. All the obtained cross-linked PAMAM at different ratios were analyzed as reported in Table 2. From these preliminary results, polymer **1**(1-1) showed the lowest size and PDI, confirming this sample as the most suitable candidate for gene delivery experiments. Moreover, for polymers **1**(2-1) and **1**(1-2), we measured an increase in size after complexation with the genetic materials. For this reason and being the polymer obtained with the highest yield and with the sharper and more intense fluorine signal, cross-linked polymer **1**(1-1) was selected as the lead candidate for a deep investigation, starting from the evaluation of the surface charge with (w/) and without (w/o) gene cargo and the size after gene complexation. As highlighted in Table 2 after complexation with the genetic cargo, we observed a notable reduction in the size of the dendriplex, probably due to both the electrostatic interactions between the positive charges of cross-linked dendrimer **1**(1-1) and the negative charges in the nucleic acid and hydrophobic interactions between the fluorinated moieties of **1**(1-1) and the pDNA backbone. Moreover, a reduction of the ξ-potential of the dendriplexes was measured, underlining the occurred electrostatic complexation. Notably, dendriplexes with a size of approximately 200 nm have been shown to be suitable for gene transfection.^41,42^

**Table 2:** Intensity-Weighted Mean Hydrodynamic Size (DH), Polydispersity Index (PDI), and ξ-Potential of the Fluorinated Dendrimers Obtained without (w/o) and with (w/w) the Genetic Cargo at 2.1 w/w (Weight Dendrimer/Weight Plasmid)

molar ratio PAMAM G2:diacrylate	*D*_H_ w/o [nm]	PDI w/o [−]	ξ-potential w/o [mV]	*D*_H_ w/ [nm]	PDI w/ [−]	ξ-potential w/ [mV]
2:1	372.6 ± 40.6	0.36 ± 0.01		532.0 ± 94.9	0.23 ± 0.1	
1:1	187.0 ± 1.6	0.28 ± 0.02	21.0 ± 1.8	141.0 ± 0.4	0.32 ± 0.02	14.2 ± 1.2
1:2	221.0 ± 28.0	0.56 ± 0.27		321.0 ± 42.9	0.63 ± 0.5	
1:4	218.7 ± 47.9	0.36 ± 0.35		157.4 ± 21.9	0.28 ± 0.1	

Agarose gel analysis (Figure 5) showed that cross-linked polymer **1**(1-1) effectively complexed pDNA at various weight-to-weight (w/w) ratios (1.0, 2.1, and 4.2), as evidenced by the retardation of pDNA migration compared to free pGFP (second lane).

**Figure 5:**
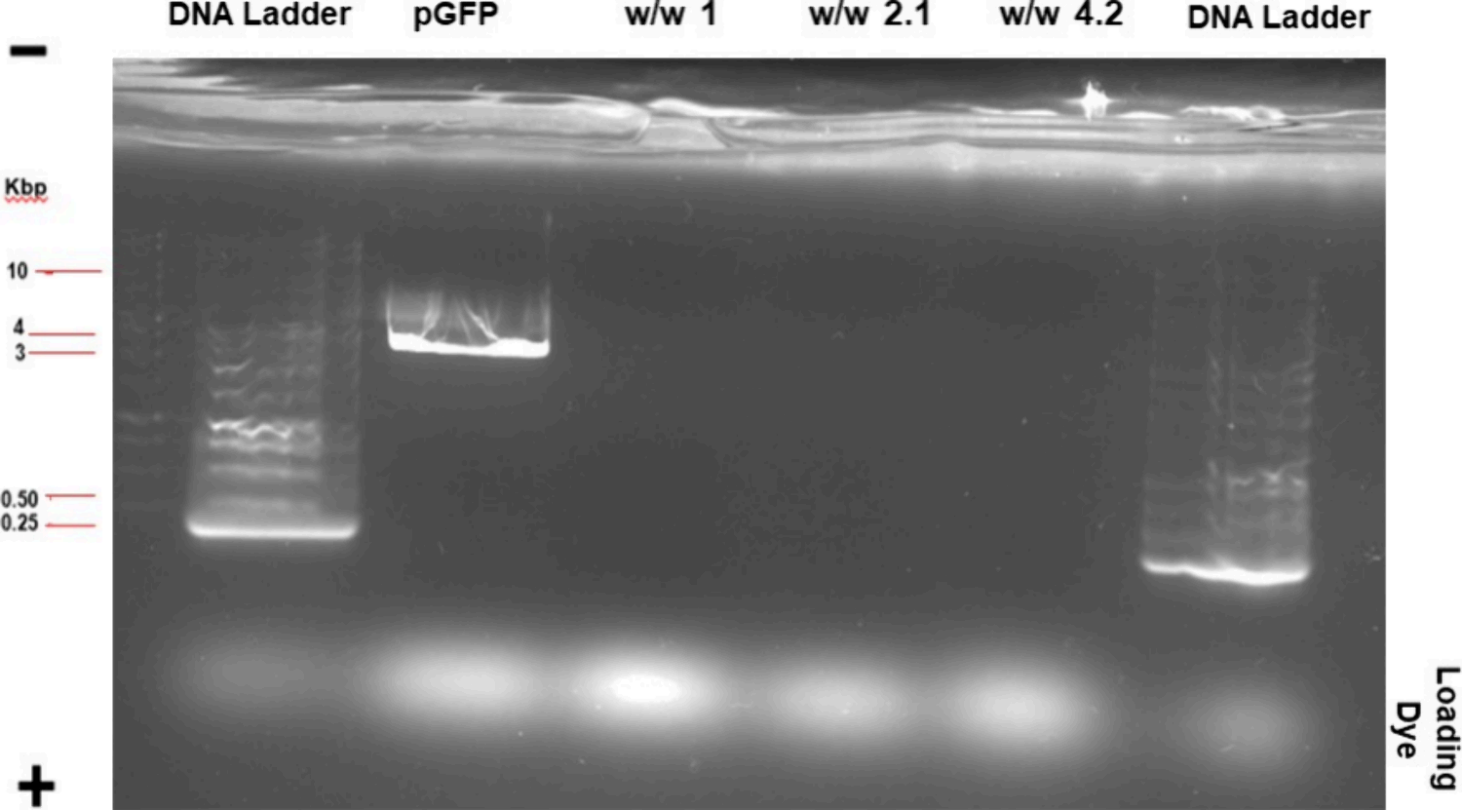
Agarose gel analysis of the plasmid complexed by fluorinated cross-linked polymer **1**(1-1) at different w/w ratios. The complexes derived from 1 at different w/w ratios were loaded and compared to DNA ladders (first and last lane) and free DNA (pGFP, second lane). The in-well DNA staining indicates good complexation of the synthesized systems, whereas free DNA migration along the gel is observed. As the free plasmid has not been linearized, two slight bands are visible at an apparent higher weight due to supercoiling of the nucleic acid.

### GSH-Responsive Behavior of Fluorinated Cross-Linked PAMAM G2

The GSH-responsive behavior of **1**(1-1) was investigated through DLS and agarose gel electrophoresis. The cross-linked polymer **1**(1-1) was dissolved in a 10 mM GSH solution for 48 h at 37 °C to simulate the intracellular environment of tumor cells. As shown in Figure 6, we detected a decrement in size over the first 24 h, indicating breakdown of disulfide bridges within the cross-linked polymer in the presence of GSH and the consequent increase in the number of particles detected by the instrument.

**Figure 6:**
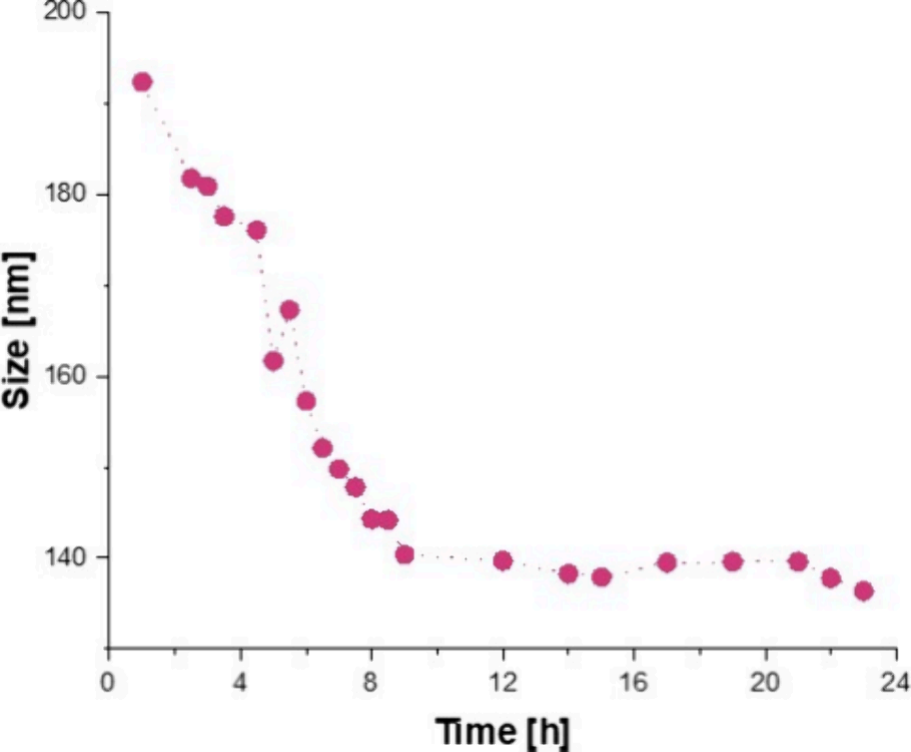
Degradation kinetic of **1**(1-1) in GHS-rich conditions is reported as size versus the time in hours.

Agarose gel retardation was done to investigate the GHS-responsive behavior of the corresponding polyplex with pDNA. Accordingly, the assay was performed comparing the behavior of fluorinated cross-linked PAMAM G2 **1**(1-1) complexed with plasmid and incubated for 48h in GSH-rich solution to that of fluorinated cross-linked PAMAM G2 **1**(1-1)/plasmid complexes in aqueous solution without GSH (Figure 7).

**Figure 7:**
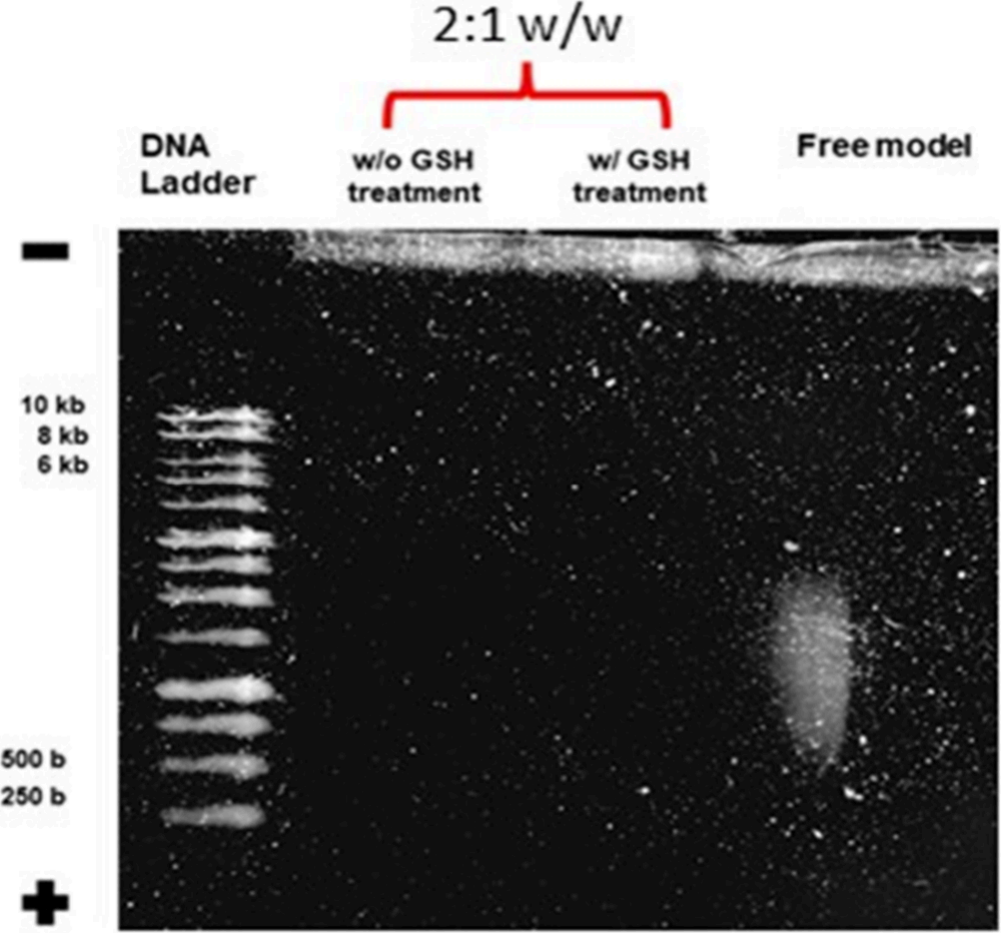
Agarose gel analysis of the herring sperm DNA complexed by cationic fluorinated cross-linked dendrimers **1**(1-1) treated (w/) or not treated (w/o) with 10 mM GSH aqueous solution. In the first and last well, the DNA ladder and free DNA were loaded, respectively. The stain of DNA with Diamond dye indicates the good complexation of the synthesized system, whose complexed DNA, showed as a bright fluorescent blot, remains in the loaded wells, whereas free DNA migration along the gel is observed.

The complexation ability is still maintained after treatment with a GSH-rich solution (no plasmid migration was observed), probably indicating that also after the disulfide bond cleavage the cationic dendritic structures are still able to maintain the electrostatic interaction with the genetic cargo. This result is not surprising since it is reported that PAMAM G2 alone is able to complex pDNA at N/P = 1.^43^

### In Vitro Cytotoxicity and Transfection Efficiency of pDNA/Fluorinated Cross-Linked PAMAM G2 Complex

Preliminary in vitro TE and cytotoxicity experiments on the nontumoral human umbilical vein endothelial cells (HUVEC) were performed utilizing cross-linked PAMAM G2 **1**(1-1) and pDNA complexes with a 2.1 w/w ratio (**1**(1-1)/plasmid). Non-cross-linked PAMAM G2 and 25 kDa branched polyethylenimine (bPEI), which is considered the golden standard in transfection assays with polymeric nonviral vectors, were selected as references, along with HUVEC without transfecting agent as a negative control. The cross-linked PAMAM G2 **1**(1-1) dendriplex successfully transfected cells after 48 h (Figure 8A,B). This indicates that the dendriplex is effective in crossing the cell membrane and releasing the cargo inside the cell, leading to a significant number of transfected cells, approximately 30%. It is worth noting that the performance of **1**(1-1) is notably better compared to undecorated PAMAM G2 and 25 kDa bPEI. Compared to the latter, the cross-linked PAMAM G2 **1**(1-1) could increase the transfection efficiency by a factor of 2, which is a remarkable result in the direction of efficient nonviral gene delivery systems. These promising results were complemented with the analysis of cytotoxicity and investigated via the MTT assay after 48 h. This delineates higher viability of treated cells with fluorinated vector **1**(1-1) with and without pGFP cargo compared to PAMAM G2 and 25 kDa bPEI, which showed higher cytotoxicity (see Figure 10).

**Figure 8:**
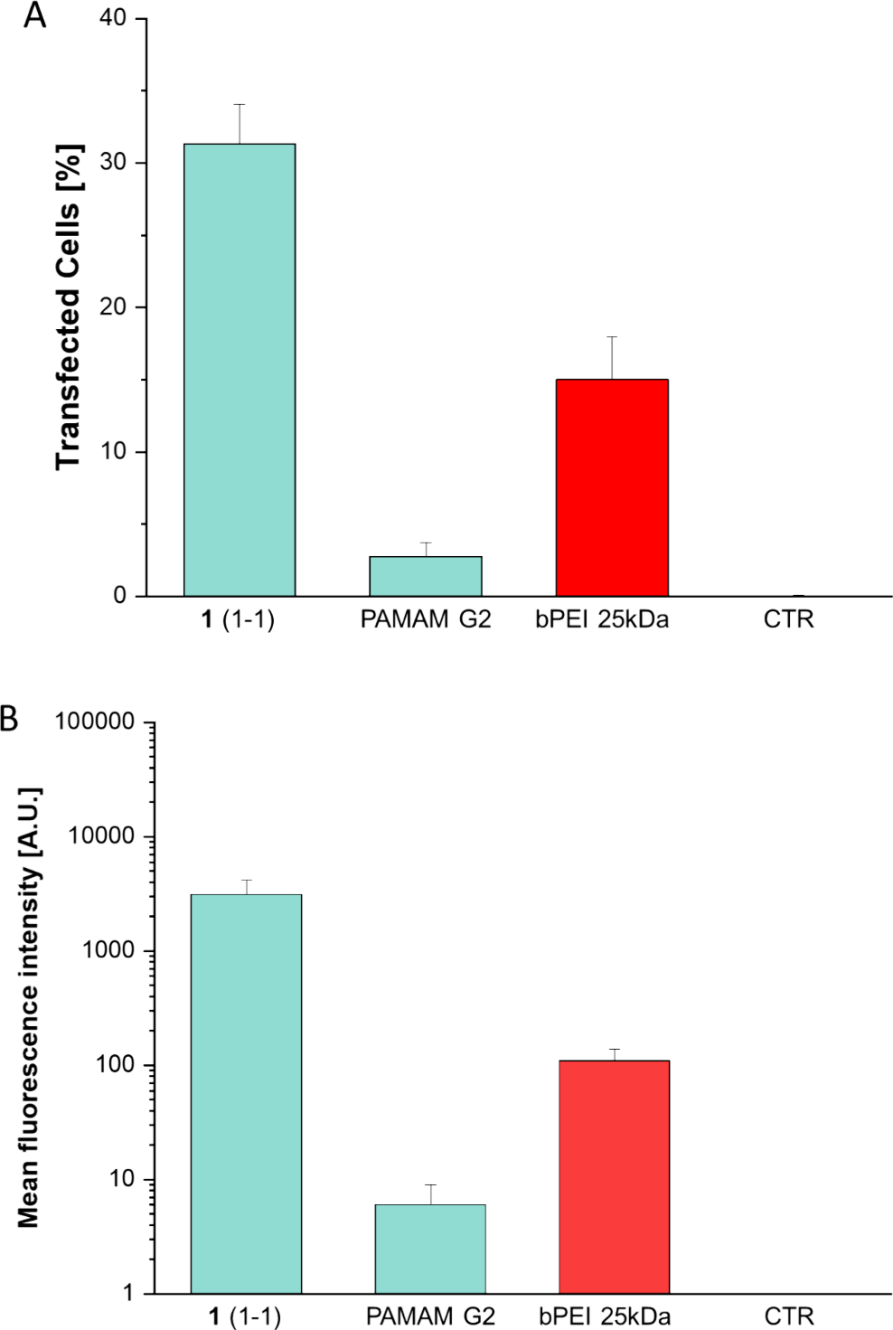
TEs of fluorinated cross-linked PAMAM **1**(1:1) compared to PAMAM G2 and bPEI 25 kDa (w/w fixed to 2.1) in HUVECs. Panels (A) and (B) depict TE tests at w/w 2.1, as percentage of transfected cells and fluorescence intensity measured via ImageJ software, respectively, after 48h. For negative control (CTR), HUVECs without transfecting agent were considered. Error bars are standard deviation and statistical significance is *p* < 0.001 for all dendrimers compared to the positive control bPEI 25 kDa.

These preliminary studies on HUVECs not only showed the extremely low cytotoxicity of **1**(1-1) but also underscored its noteworthy high internalization capacity, presenting the obtained fluorinated system as a promising candidate for further exploration in nonviral vector applications on cancer cells for innovative and theranostic cancer therapy. Accordingly, in vitro TEs and cytotoxicity assays on a tumoral cell line (HeLa) were performed to investigate the possible enhanced TE due to the GHS-responsivity of vector **1**(1-1). In this direction, for the sample **1**(1-1), we investigated the TE at different ratios between the nanoclusters and the pDNA, namely, w/w 1.0, 2.1, and 4.2 (Figure 9A,B).

**Figure 9:**
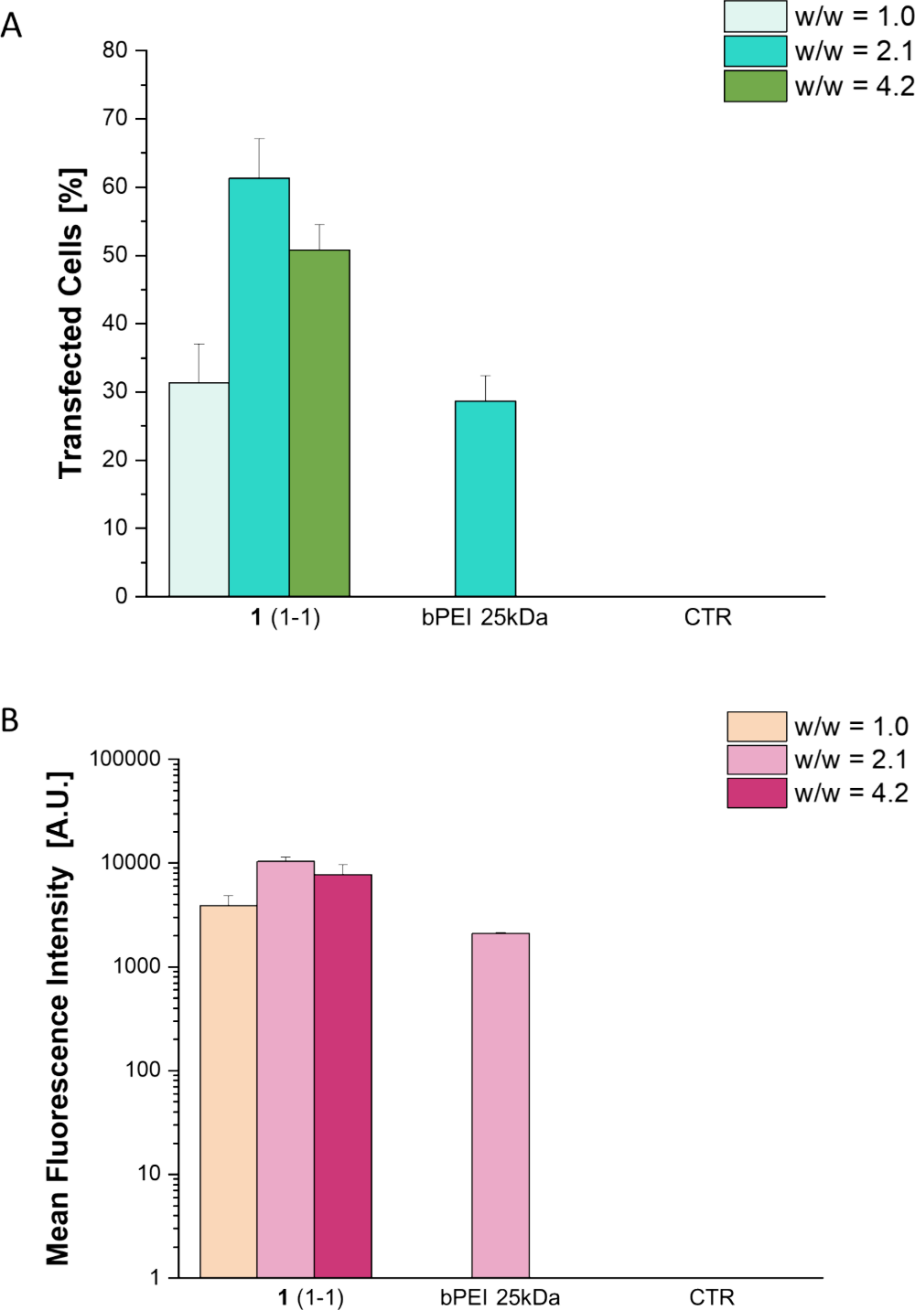
TEs of polyplexes formed with fluorinated cross-linked PAMAM **1**(1:1) and pDNA at different w/w ratios compared to bPEI 25 kDa (w/w fixed to 2.1) in HeLa cells. Panels (A) and (B) depicts TE tests as percentage of transfected cells and fluorescence intensity measured via ImageJ software, respectively, after 48h. For negative control (CTR), HeLa cells without transfecting agent were considered. Error bars are standard deviation, and statistical significance is *p* < 0.001 for all dendrimers compared to the positive control bPEI 25 kDa.

Gratifyingly, the ability to transect HeLa cells of **1**(1-1) was much higher compared to the TE obtained with HUVECs at the same w/w ratio, with a w/w ratio of 2.1 being the most efficient. Indeed, at w/w ratio 2.1, the cells transfected were around 60%, circa twice the TE obtained with the HUVECs, demonstrating that the polymer degradation in a GSH-rich environment is important for an enhanced pDNA release. Furthermore, at all w/w ratios, the TEs of **1**(1-1) in cancer cells were higher than those of golden standard 25 kDa bPEI. Moreover, **1**(1-1) polyplex resulted to have negligible cytotoxicity, being less cytotoxic than 25 kDa bPEI as evidenced in Figure 10.

**Figure 10:**
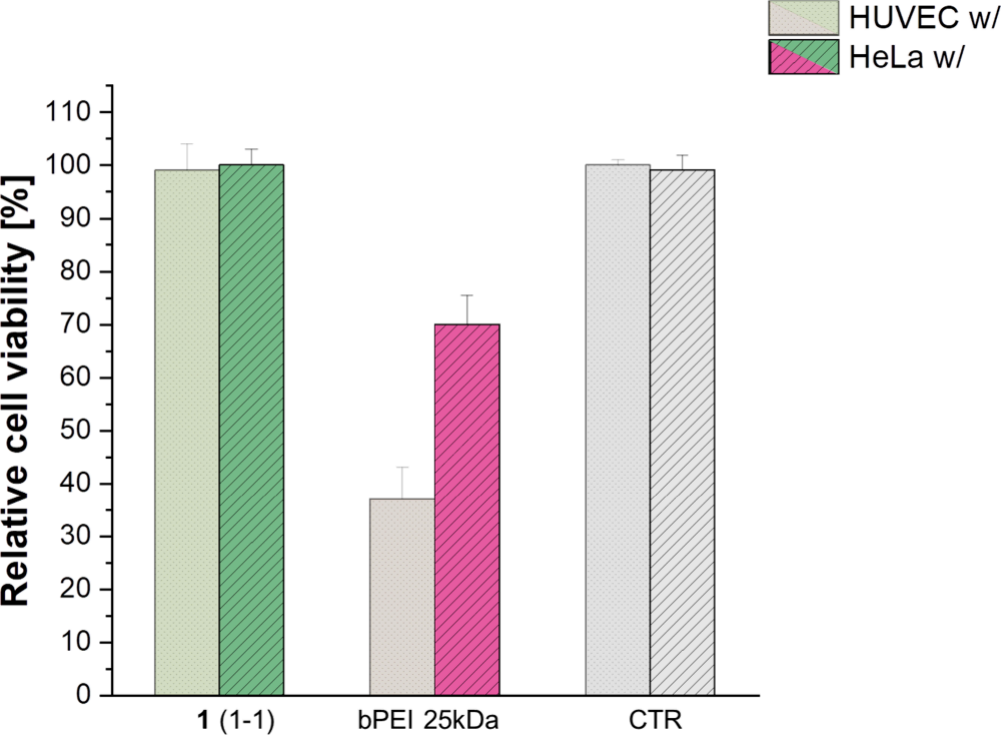
Cytotoxicity expressed as cell viability relative to the control for cross-linked polymer **1**(1-1) and bPEI 25 kDa in HUVECs and HeLa cells. Error bars are standard deviation, and statistical significance is *p* < 0.001 for all dendrimers compared to the positive control bPEI 25 kDa.

A comparison between the TEs of fluorinated cross-linked polymer **1**(1-1) in both HUVECs and HeLa cells is also shown in Figure 11, which presents the fluorescent micrographs of cells transfected at w/w 2.1 along with those transfected with bPEI 25 kDa and compared to a negative control showing untreated cells. The density and intensity of fluorescent cells are significantly higher for **1**(1-1)/pDNA in HeLa cells than in HUVECs and even higher than those for bPEI 25 kDa/pDNA, confirming that **1**(1-1) is a valuable redox-responsive gene delivery vector and showcasing its therapeutic potential for tumor treatment.

**Figure 11:**
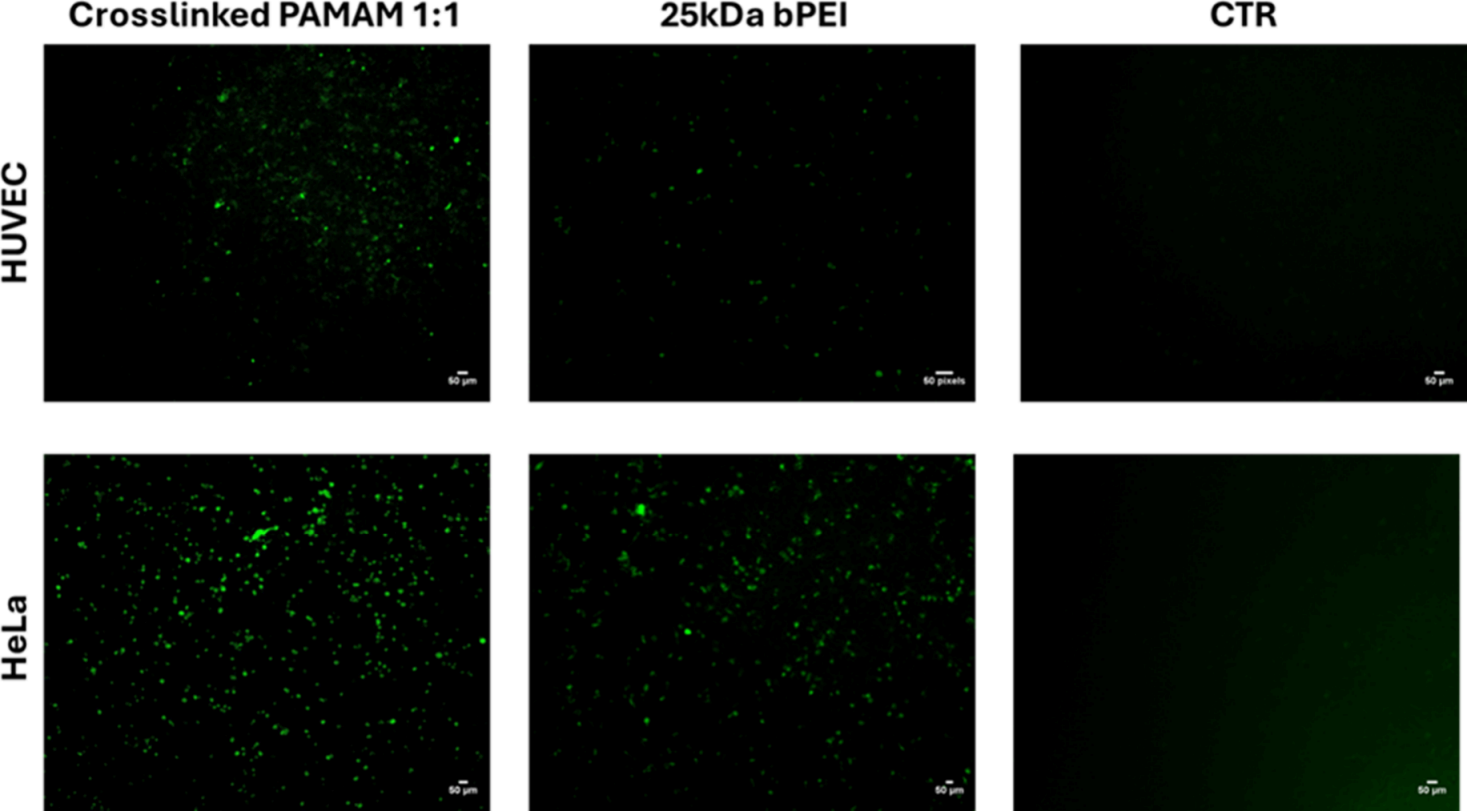
Transfected cell micrographs acquired with Celena S microscopy (filter cube GFP) after 48 h (T48) from dendriplexes added to HUVECs and HeLa cultures. The images depict cell cultures treated with fluorinated cross-linked PAMAM **1**(1:1) compared to bPEI 25 kDa; the emitted green fluorescence is related to the GFP expressed by the transfected cells. For negative control (CTR), HUVECs and HeLa without any transfecting agent were considered.

## Conclusions

One of the most challenging issues hampering the translation to the clinic of PAMAM dendrimers as efficient and safe gene delivery vectors relies on the paradox of increasing transfection efficiency and cytotoxicity with the generation of the dendrimer. One possible strategy to solve this problem is to increase the efficiency of low-generation PAMAM dendrimers, which are both low cost and relatively well tolerated by chemical functionalization. In particular, the cross-linking of low-generation PAMAM dendrimers with redox-responsive cross-linkers is a particularly intriguing strategy as it can impart controlled release of the genetic cargo after polyplex internalization and at the same time copolymer degradation into less cytotoxic monomers. In the present study, we presented the synthesis, for the first time, of a redox-responsive cross-linker containing two equivalent tfm groups. The cross-linking with low-generation PAMAM G2 dendrimer occurs straightforwardly through “click” Michael addition, producing fluorinated polymers with sharp and intense fluorine nuclear magnetic resonance signals and favorable relaxation parameters, thus suitable for an implementation as ^19^F MRI tracers. The multifunctional cross-linked polymer obtained by reacting an equimolar ratio of PAMAM G2 and fluorinated cross-linker resulted to be the most interesting in terms of ^19^F NMR properties and biophysical features, showing also higher transfection efficiency and safer profile than the monomer PAMAM G2 and even than the gold standard bPEI 25 kDa. The transfection efficiency was higher in cancer cell lines (HeLa) than in nontumoral HUVECs, demonstrating that the selective cleavage of the disulfide bridge in the cross-linker in a GSH-rich environment is effective in enhancing the pDNA release. Overall, these findings demonstrate that the fluorinated bioreducible cross-linking strategy decouples the traditional link between high dendrimer generation and transfection efficiency. This enables low-generation PAMAM G2 dendrimers to achieve strong transfection performance, particularly in cancer cells, along with ^19^F MRI trackability and minimal cytotoxicity. Additionally, the fluorinated stimuli-responsive cross-linker can be applied to other low molecular weight polymers, such as linear and branched PEI and poly­(l-lysine), among others, offering a versatile and reliable platform for creating efficient, safe, and trackable multifunctional gene delivery systems.

## Supplementary Material



## Notes

Experimental procedure and chemical characterization of model compound **3**; ^1^H, ^13^C, and ^19^F NMR spectra of CBtfmA **2** and model compound **3**; copies of ^1^H NMR and ^19^F NMR spectra of copolymers **1**; kinetic cross-linking reaction study via ^19^F NMR and DLS (PDF)
