# Supplementary material for: Fluorinated Redox-Responsive Cross-Linked Poly(amidoamine) G2 as Smart Theranostic Dendrimers
Source: Biomacromolecules. 2025 Aug 11;26(9):6001–14. doi: 10.1021/acs.biomac.5c00914 (PMC12421505; doi:10.1021/acs.biomac.5c00914)

# SUPPORTING INFORMATION

# Fluorinated Redox-Responsive Crosslinked PAMAM G2 as Smart Theranostic Dendrimers

Carola Romani,[§] Maria Cristina Bellucci,[¥] Maria Enrica Di Pietro,[§] Paola Gagni,[†] Mattia Sponchioni,[§,*] Alessandro Volonterio[§,*]

*Department of Chemistry, Materials and Chemical Engineering "Giulio Natta", Politecnico di Milano, via Mancinelli 7, 20131 Milano, Italy; Department of Food, Environmental, and Nutritional Sciences, Università degli Studi di Milano, via Celoria 2, 20131 Milano, Italy; Consiglio Nazionale delle Ricerche, Istituto di Scienze e Tecnologie Chimiche "Giulio Natta" (SCITEC), Via Mario Bianco 9, 20131 Milan, Italy.*

## Table of contents

| | |
|---|---|
| Pages S2 | Scheme S1. Synthesis of model compounds **3** and experimental procedure |
| Page S3-S8 | Copies of the $^1$H NMR, $^{19}$F NMR, $^{13}$C NMR and ESI MS spectra of all new compounds. |
| Pages S9-S13 | Copies of the $^1$H NMR and $^{19}$F NMR of PAMAM crosslinked **1** |
| Page S14 | Figure S1. Kinetic crosslinking reaction study via $^1$H NMR at 60 °C |
| Page S15 | Figure S2. Kinetic crosslinking reaction study via $^{19}$F NMR and DLS |
| Pages S16-S19 | Figures S3-S6. Hydrodynamic diameter distribution histograms of the crosslinked polymers without and with the genetic cargo |

**Scheme S1.** Synthesis of model compound **3**

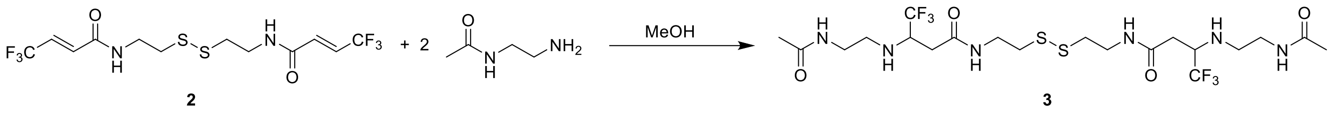

To a solution of CBtfmA **2** (100 mg, 0.25 mmol) in MeOH (2 mL) a solution of N-(2-aminoethyl)acetamide (56 mg, 0.55 mmol) in MeOH (0.5 mL) was added and the resulting solution stirred at 60 °C overnight. The solution was cooled to rt and the solvent evaporated. Model compound **3** was recovered after flash chromatography in 71 % yield (106 mg) as a white solid.

$^{1}$H-NMR (400 MHz, $CD_3OD$) δ 3.55-3.52 (m, 2H), 3.42 (t, *J* = 6.8 Hz, 4H), 3.14-3.11 (m, 8H), 2.46 (dd, *J* = 14.8 and 4.0 Hz, 2H), 2.30 (dd, *J* = 14.8 and 9.6 Hz, 2H), 1.85 (s, 6H); $^{19}$F NMR ($CD_3OD$, 376 MHz) δ -76.5 (d, *J* = 7.5 Hz); $^{13}$C NMR ($CD_3OD$, 101 MHz) δ 170.7, 168.5, 126.6 (q, *J* = 184.8 Hz), 56.6 (q, *J* = 28.3 Hz), 46.7, 39.5, 38.3, 36.9, 35.0, 21.3; ESI m/z 623.5 $[M+Na, (83)]^+$, 639.5 $[M+K, (100)]^+$; Anal. calcd. for $C_{20}H_{34}F_6N_6O_4S_2$: C, 39.99; H, 5.71; N, 13.99; found: C, 40.01; H, 5.70; N, 13.8.

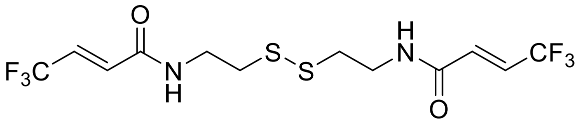

O
F3C
N
H
S
S
H
N
CF3
O


**2**

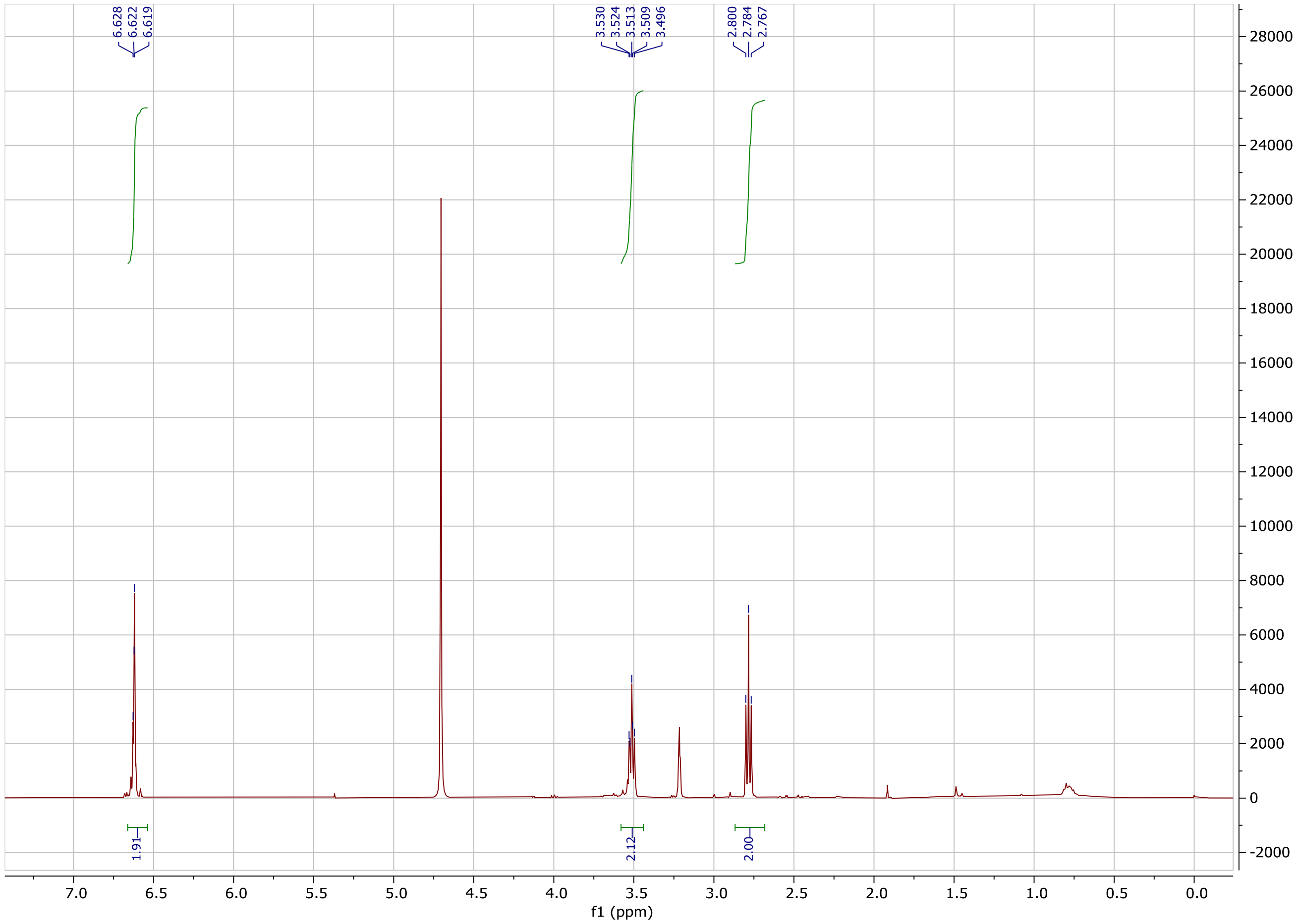

6.628
6.622
6.619
3.530
3.524
3.513
3.509
3.496
2.800
2.784
2.767
1.91
2.12
2.00
28000
26000
24000
22000
20000
18000
16000
14000
12000
10000
8000
6000
4000
2000
0
-2000
7.0
6.5
6.0
5.5
5.0
4.5
4.0
3.5
3.0
2.5
2.0
1.5
1.0
0.5
0.0
f1 (ppm)

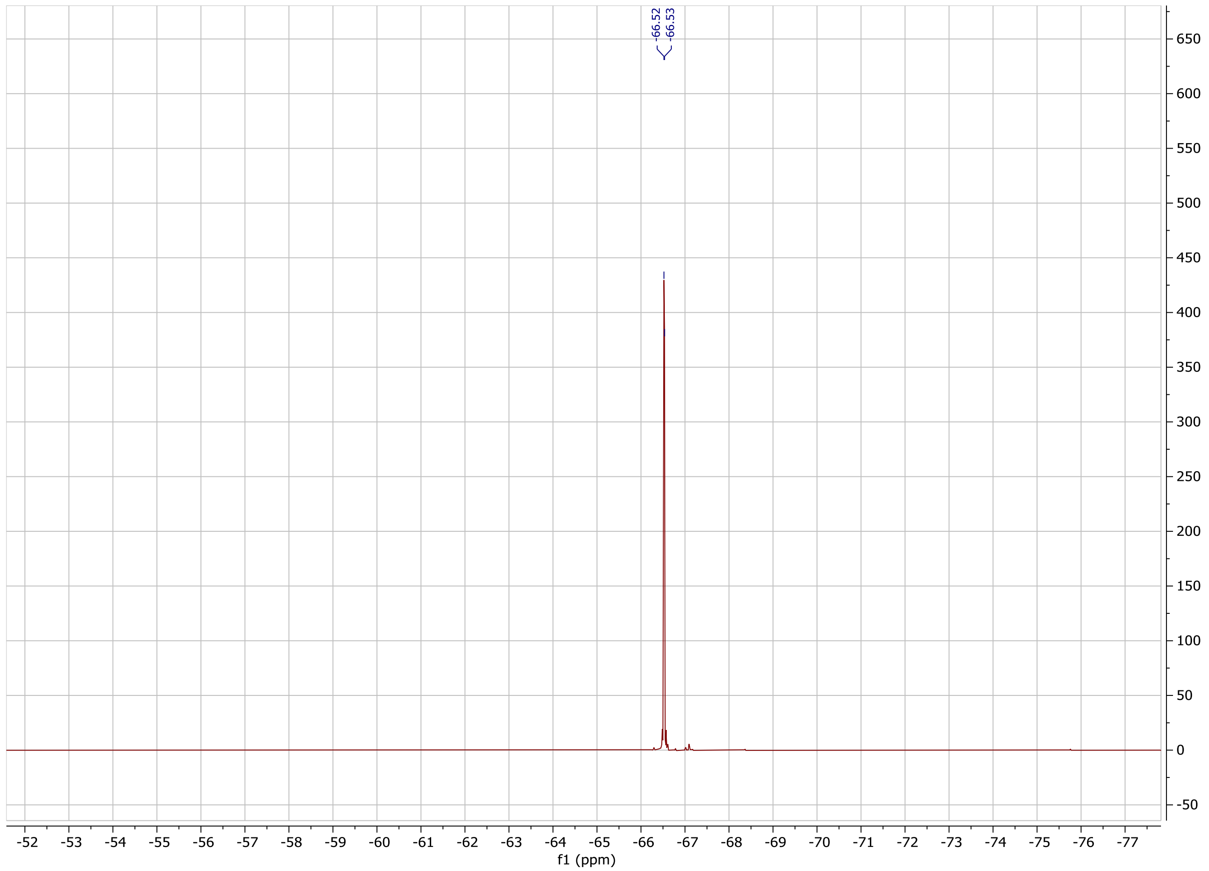
-66.52
-66.53
f1 (ppm)

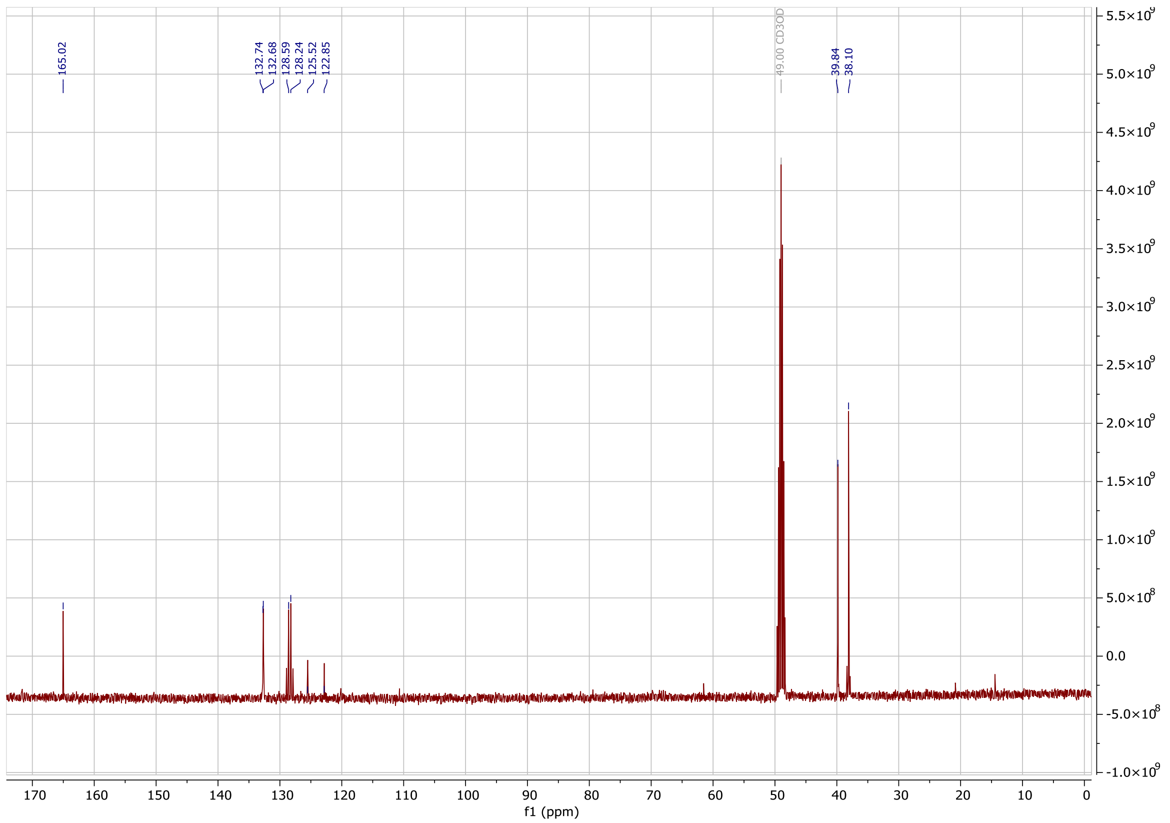
165.02
132.74
132.68
128.59
128.24
125.52
122.85
49.00 CD3OD
39.84
38.10
f1 (ppm)

## -L.G.S. - Laboratorio Grandi Strumenti - Display Report

| | | | | | |
|---|---|---|---|---|---|
| Analysis Name | av ac203.d | Acquisition Date | 04/05/22 12:46:16 | Operator | Administrator |
| Sample Name | | Method | Copy of $opt0622.MS | Instrument | esquire3000plus |
| Comment | 1 mg/mL dil 1:100 CH3CN<br>Richiedente: Caramiello | | | | |

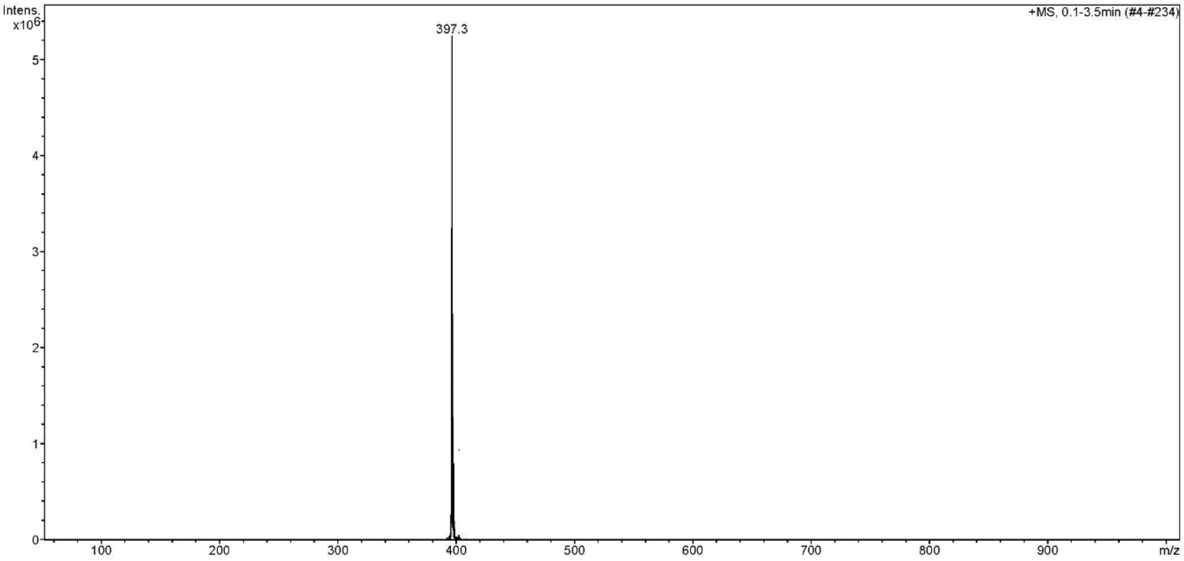


Bruker Daltonics DataAnalysis 3.1 printed: 04/05/22 13:02:54 Page 1 of 1

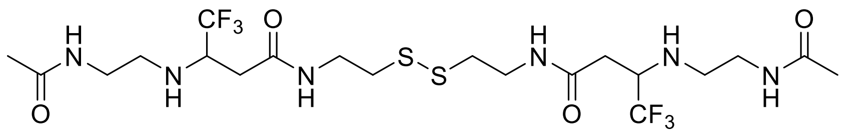
CF3
O
H
N
N
H
N
H
S
S
H
N
H
N
N
H
O
O
CF3
O

**3**

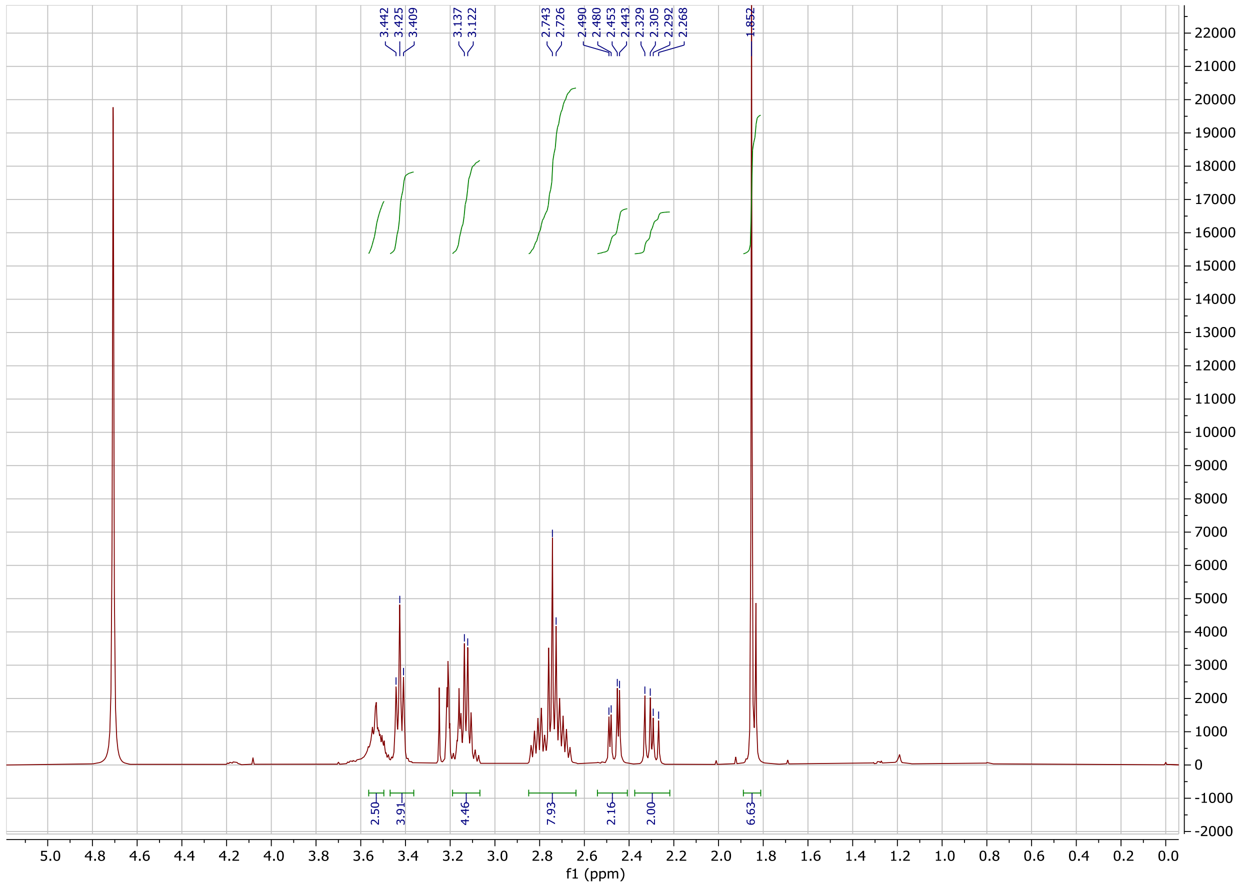
3.442
3.425
3.409
3.137
3.122
2.743
2.726
2.490
2.480
2.453
2.443
2.329
2.305
2.292
2.268
1.852
2.50
3.91
4.46
7.93
2.16
2.00
6.63
f1 (ppm)

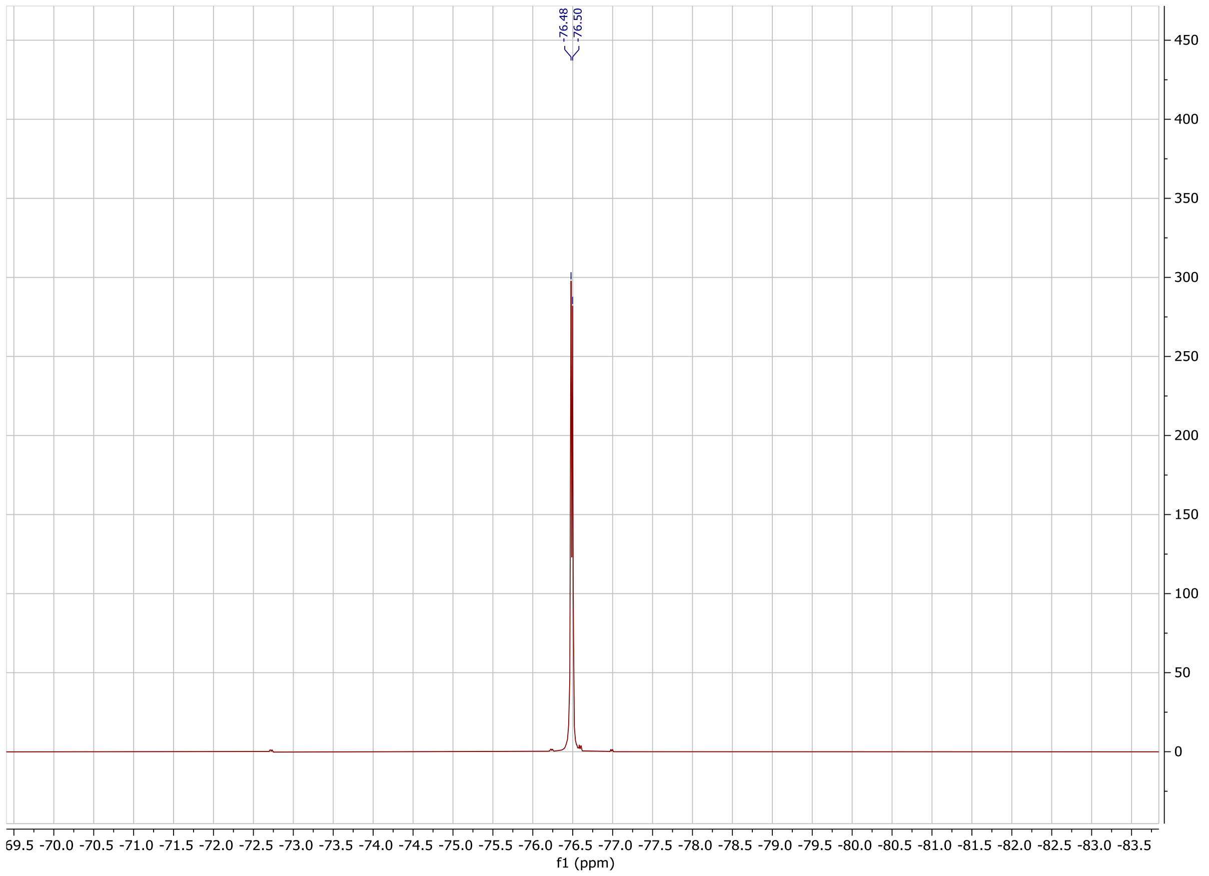
-76.48
-76.50
f1 (ppm)

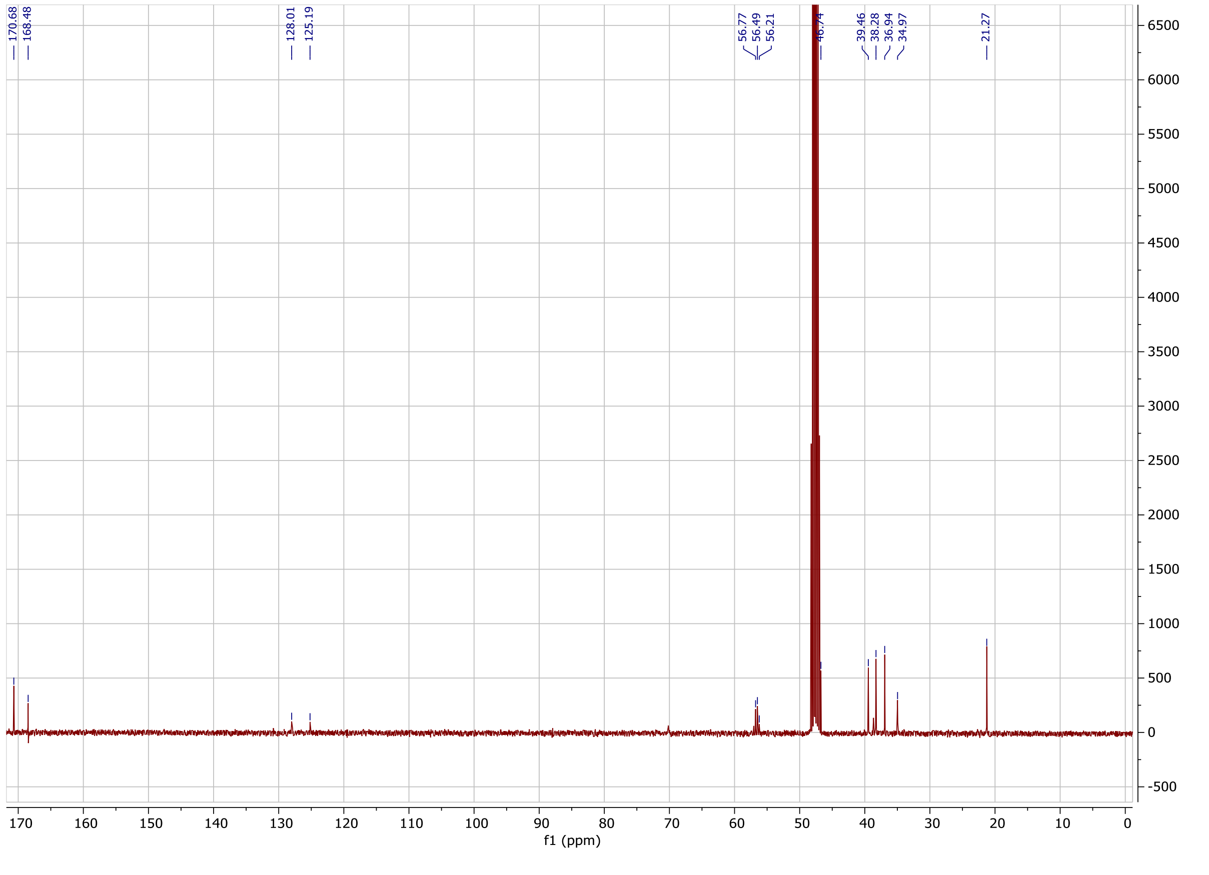
170.68
168.48
128.01
125.19
56.77
56.49
56.21
46.74
39.46
38.28
36.94
34.97
21.27
f1 (ppm)

-L.G.S. - Laboratorio Grandi Strumenti - Display Report

| | | | | | |
|---|---|---|---|---|---|
| Analysis Name | av ac224.d | Acquisition Date | 06/10/22 15:19:03 | Operator | Walter Panzeri |
| Sample Name | | Method | Copy of $opt0622.MS | Instrument | esquire3000plus |
| Comment | 1 mg/mL dil 1:100 MeOH<br>Richiedente: Caramiello | | | | |

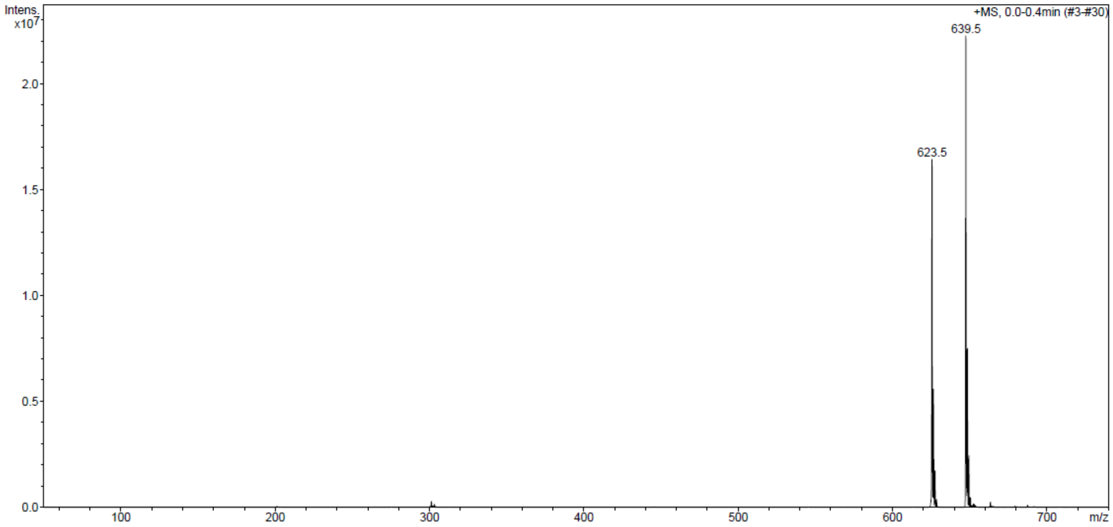


Bruker Daltonics DataAnalysis 3.1 printed: 06/13/22 14:19:30 Page 1 of 1

Fluorinated crosslinked PAMAM G2 **1**(1-1)

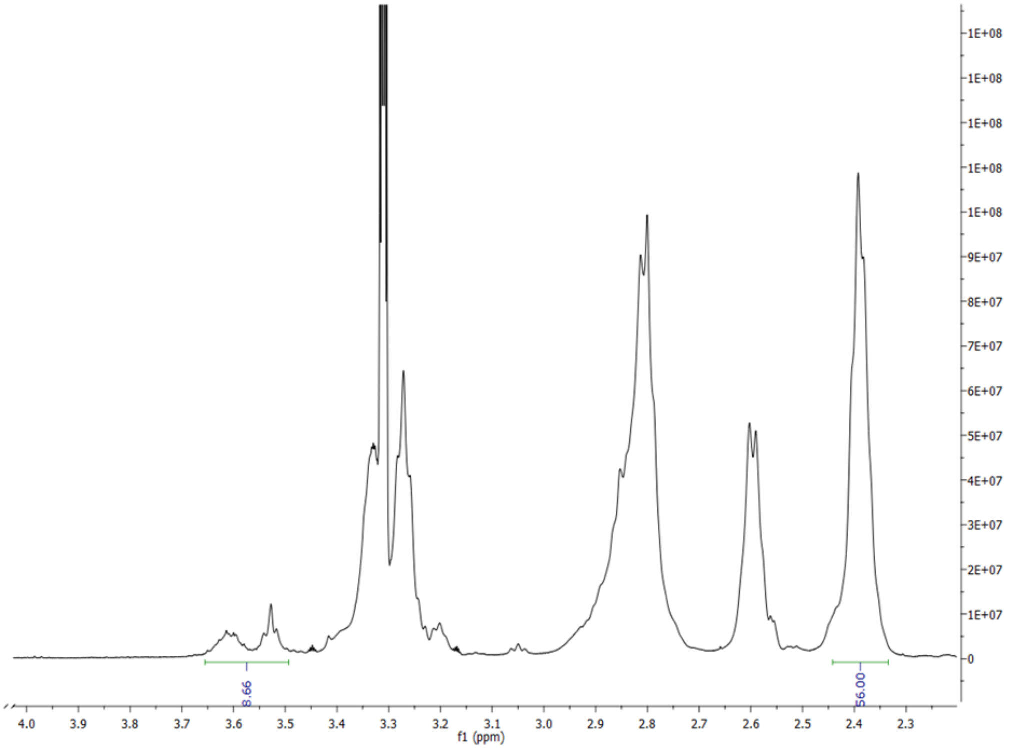


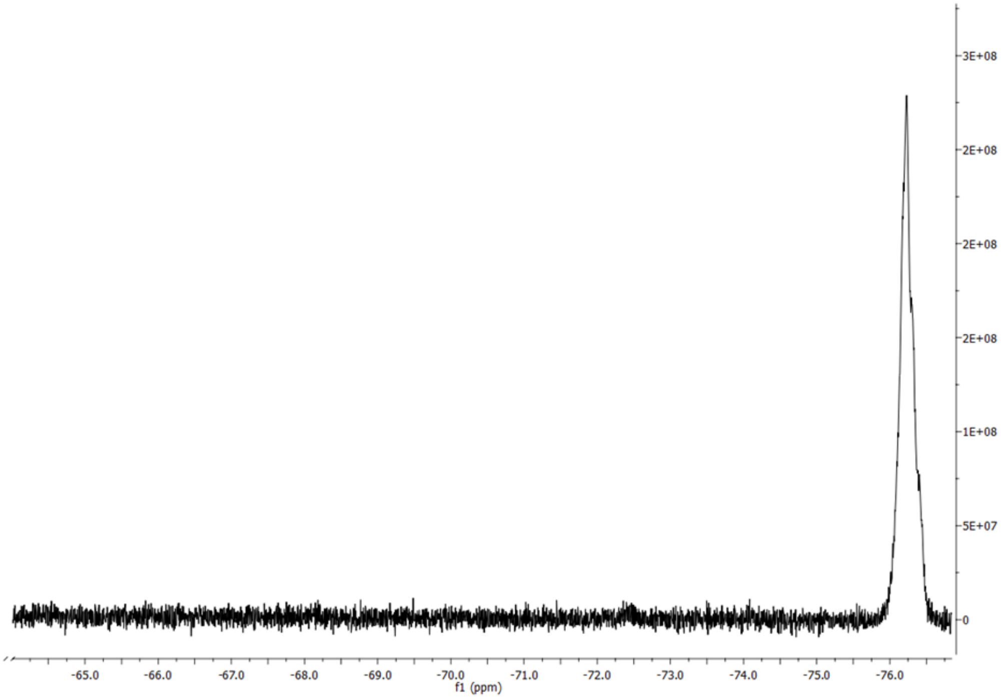

Fluorinated crosslinked PAMAM G2 **1**(1-2)

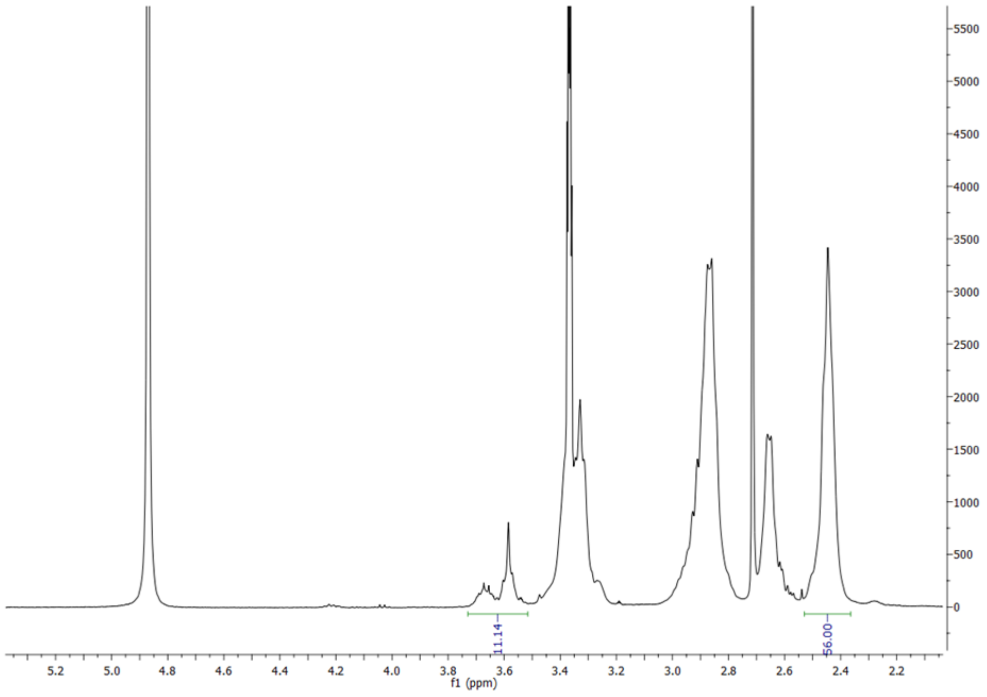


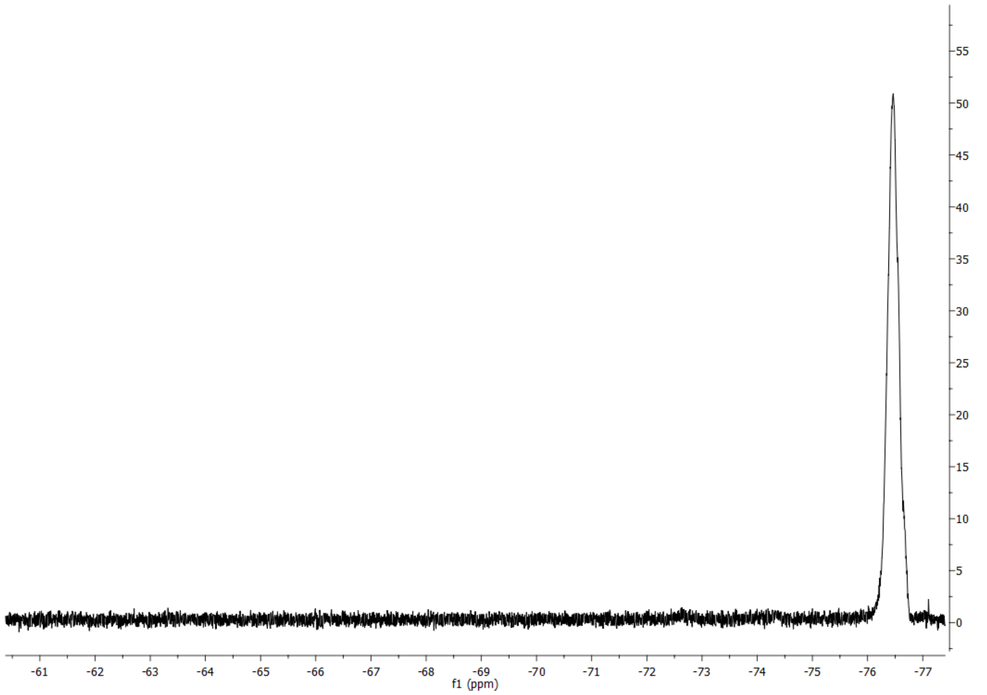

Fluorinated crosslinked PAMAM G2 **1**(1-4)

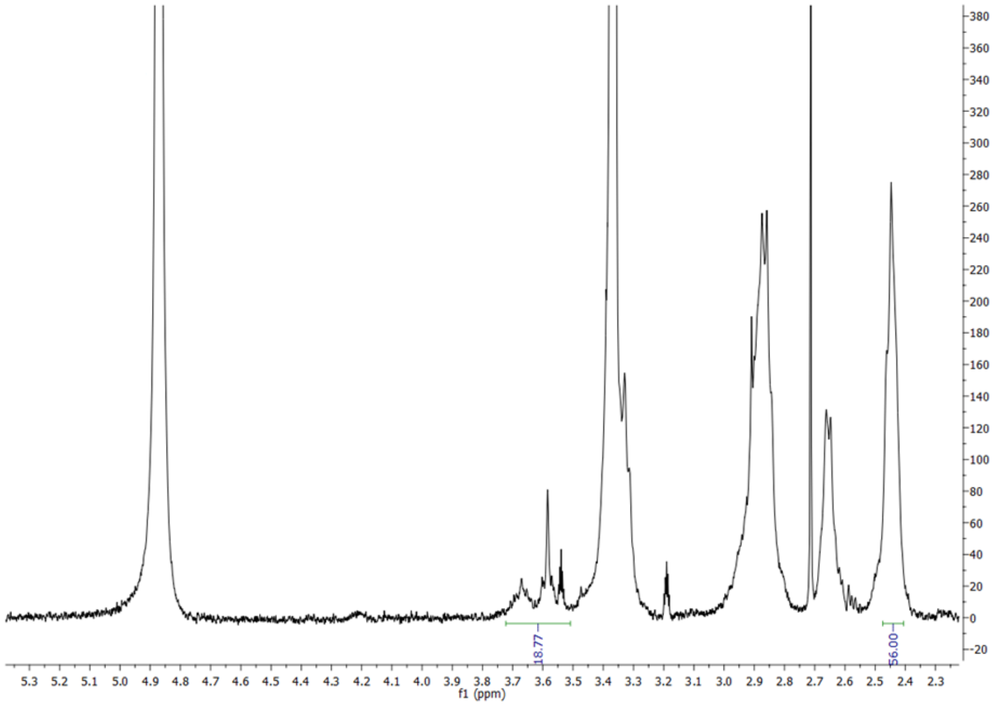


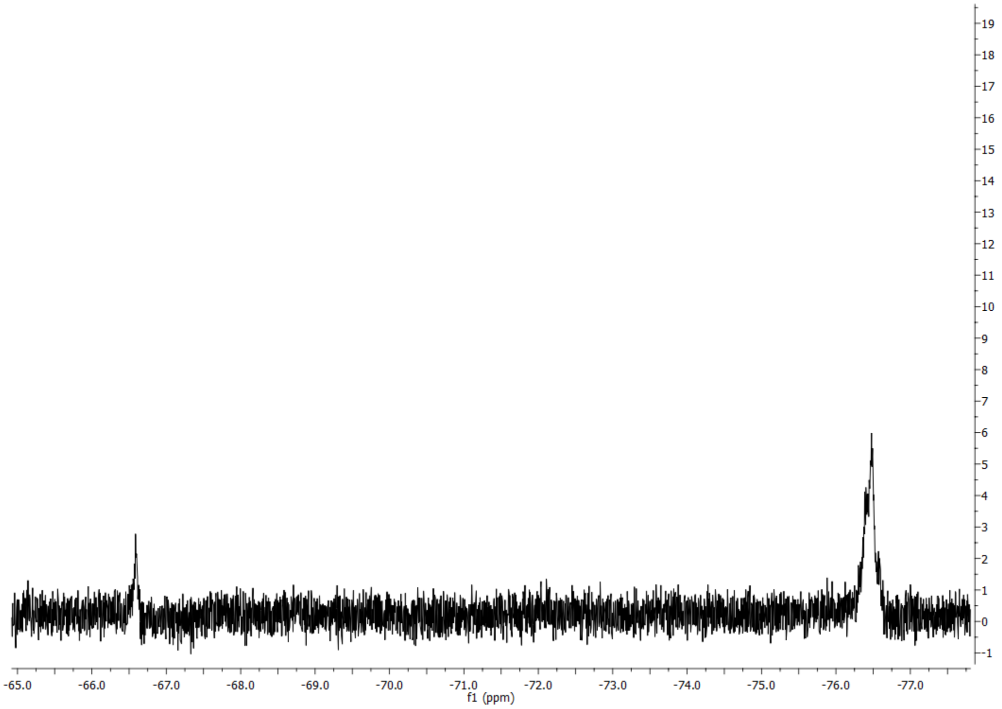

Fluorinated crosslinked PAMAM G2 **1**(2-1)

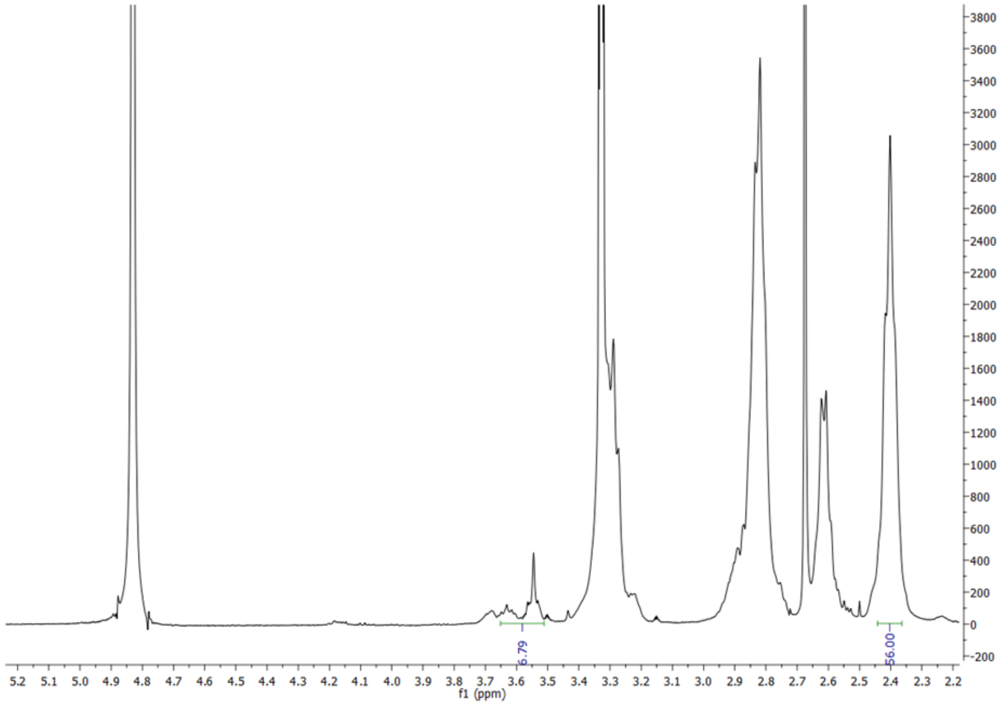


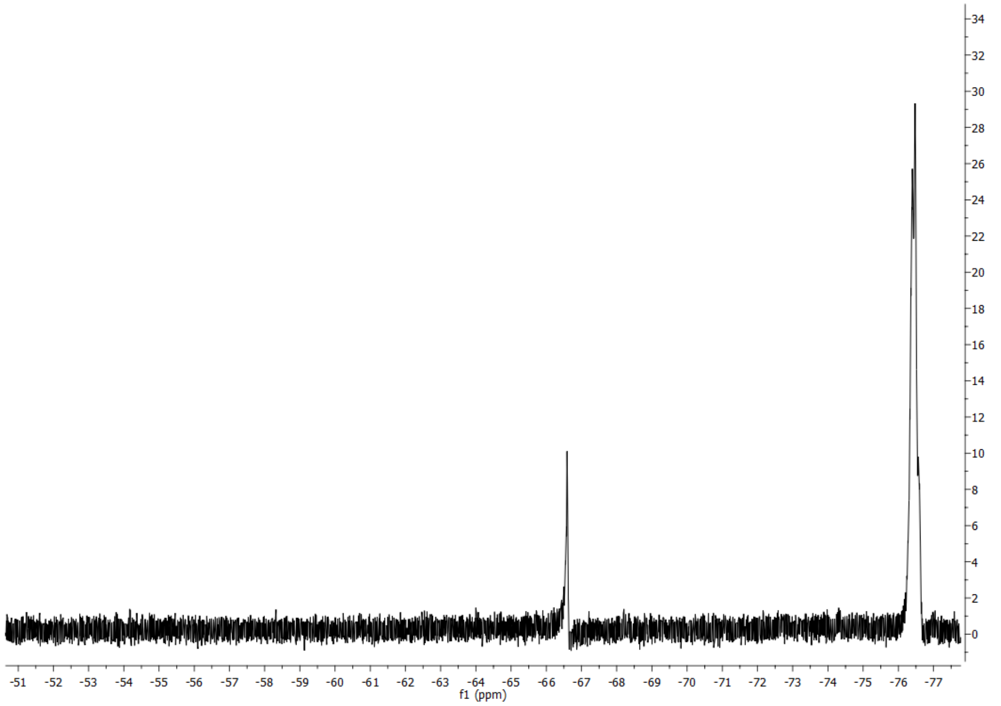

Fluorinated crosslinked PAMAM G2 **1**(4-1)

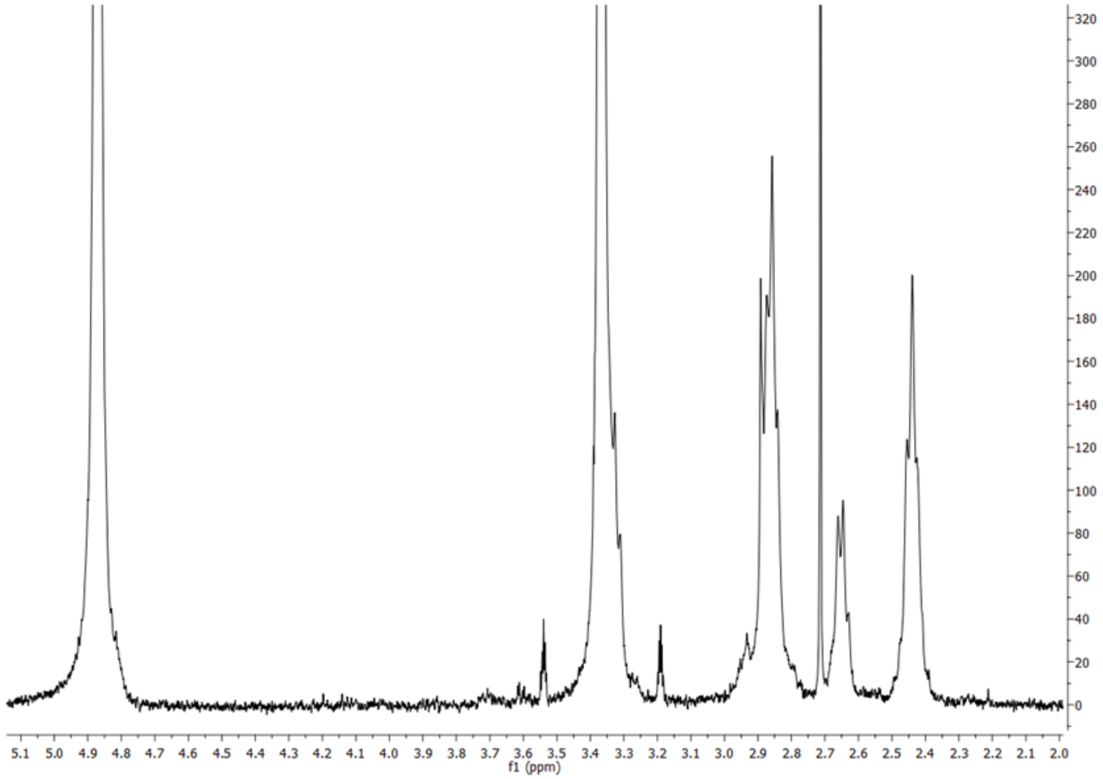


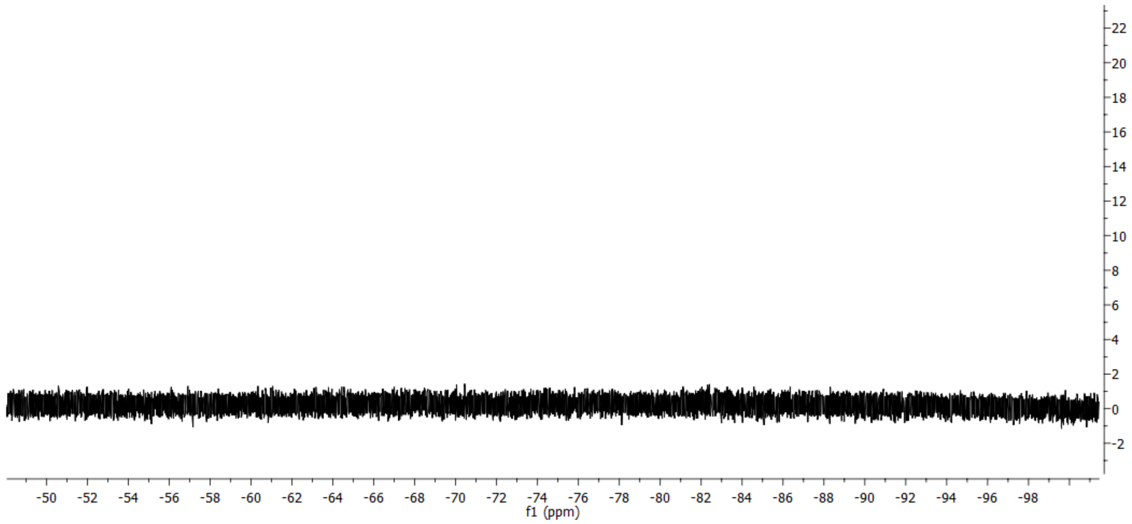

**Figure S1**. Kinetic study by $^{1}$H-NMR of crosslinked structure **1** (molar ratio 1:1) at 60 °C after 5 hours (A), 9 hours (B), 24 hours (C) and 48 hours (D).

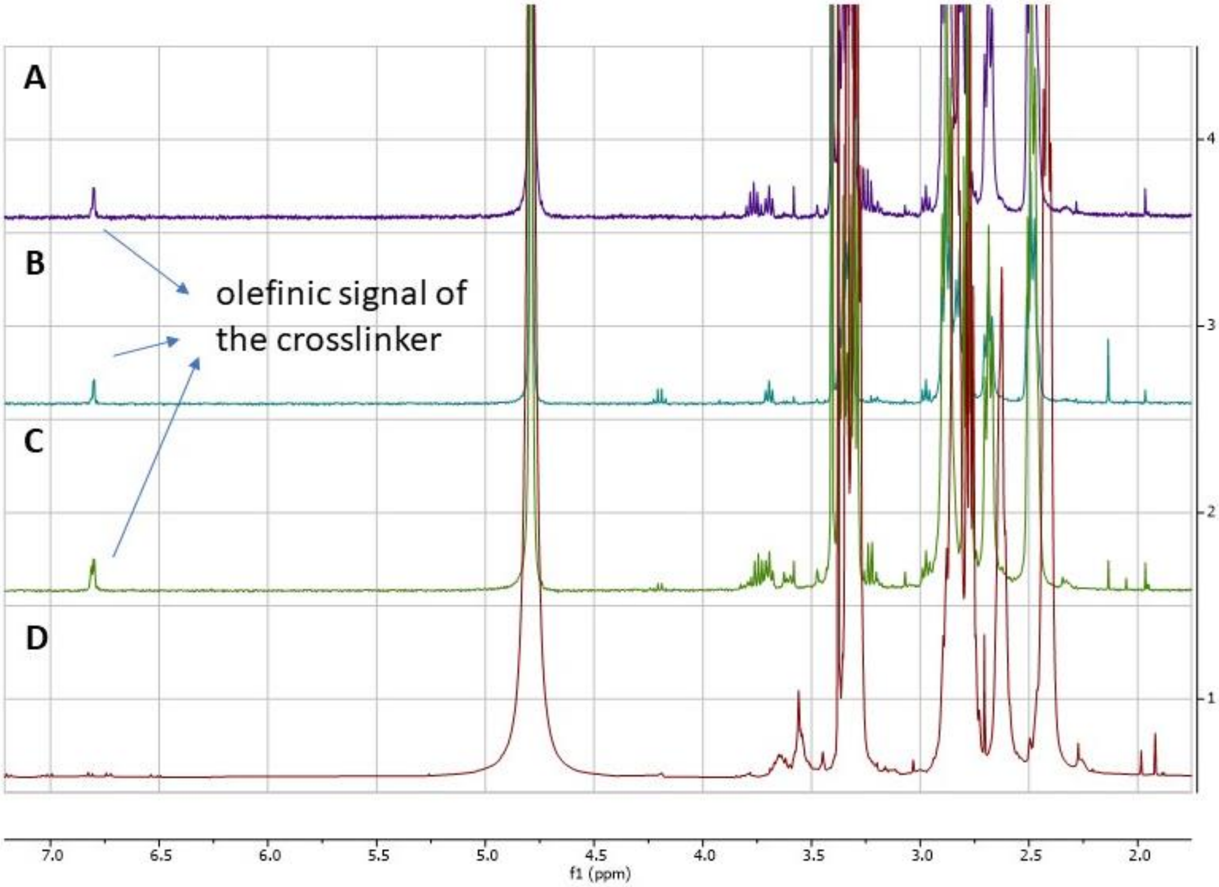

**Figure S2**. Kinetic crosslinking reaction study was performed via $^{19}$F-NMR (A) and DLS (B) analyses over 48h taking aliquots at 5h, 9h, 24h, and 48h.

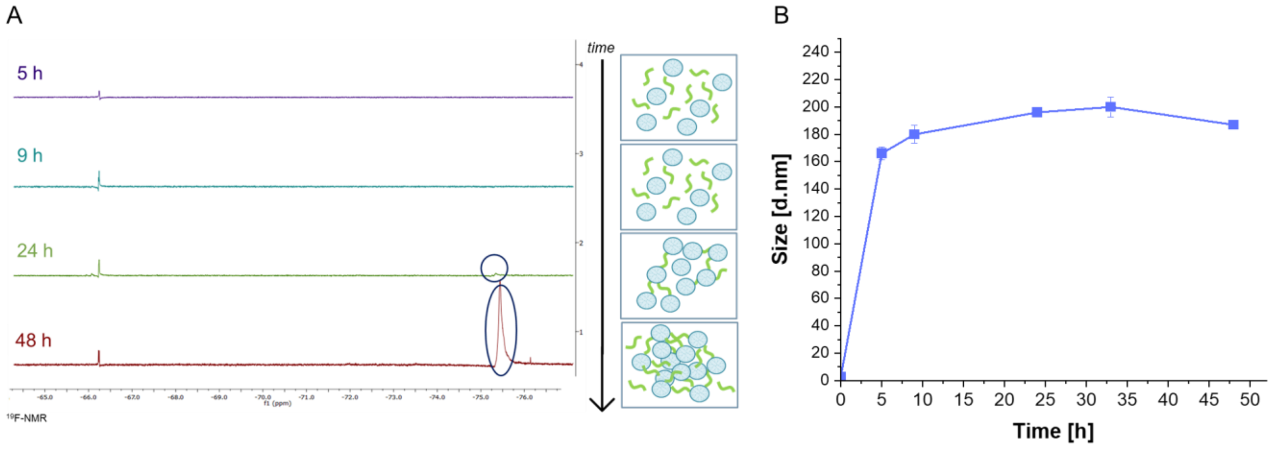

**Figure S3**. Hydrodynamic diameter distribution histograms of **1**(1-1) a) without the genetic cargo (w/o) and b) with the genetic cargo (w/)

a)

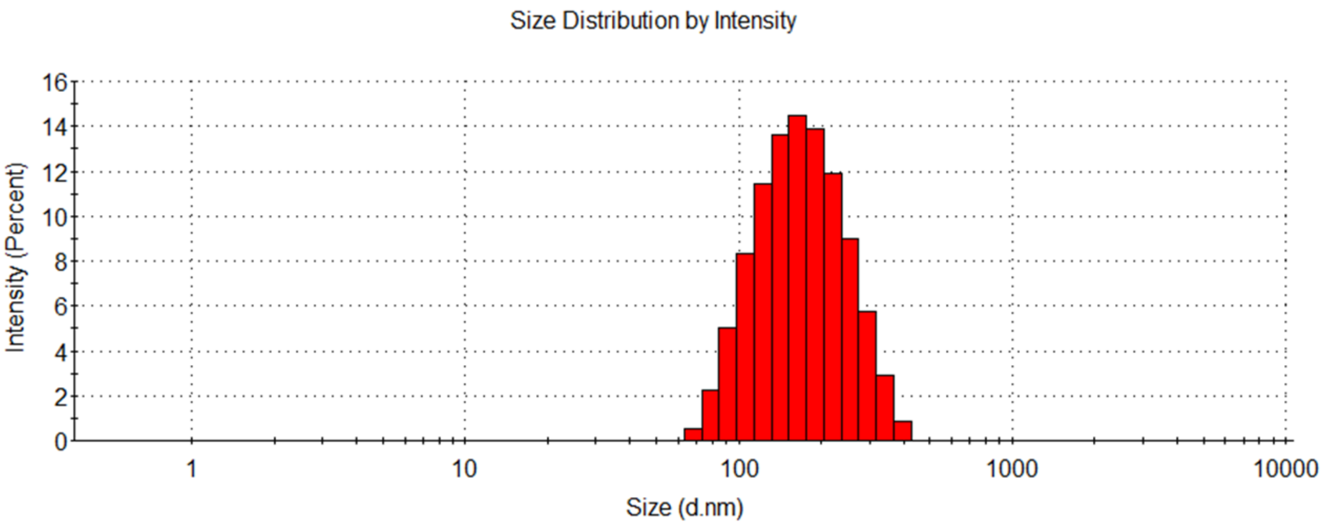


b)

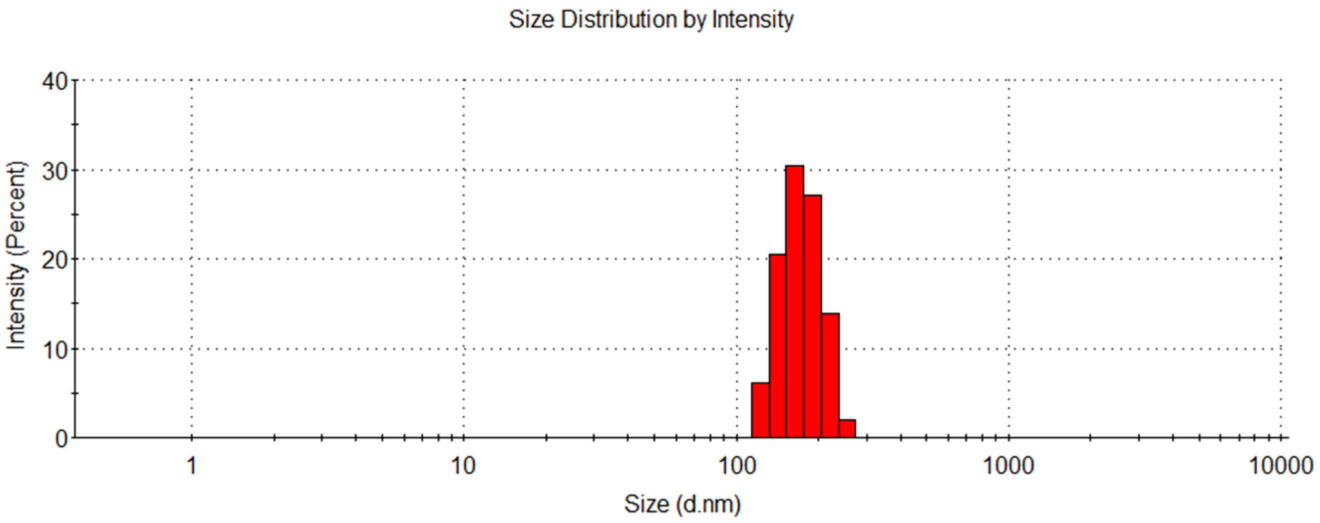

**Figure S4**. Hydrodynamic diameter distribution histograms of **1**(2-1) a) without the genetic cargo (w/o) and b) with the genetic cargo (w/)

a)

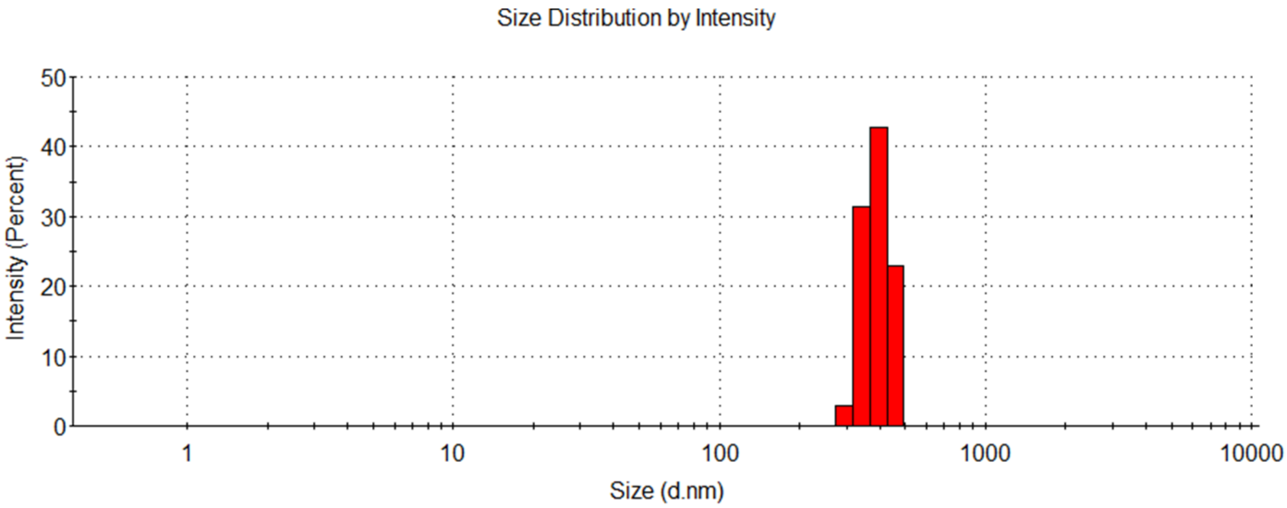


b)

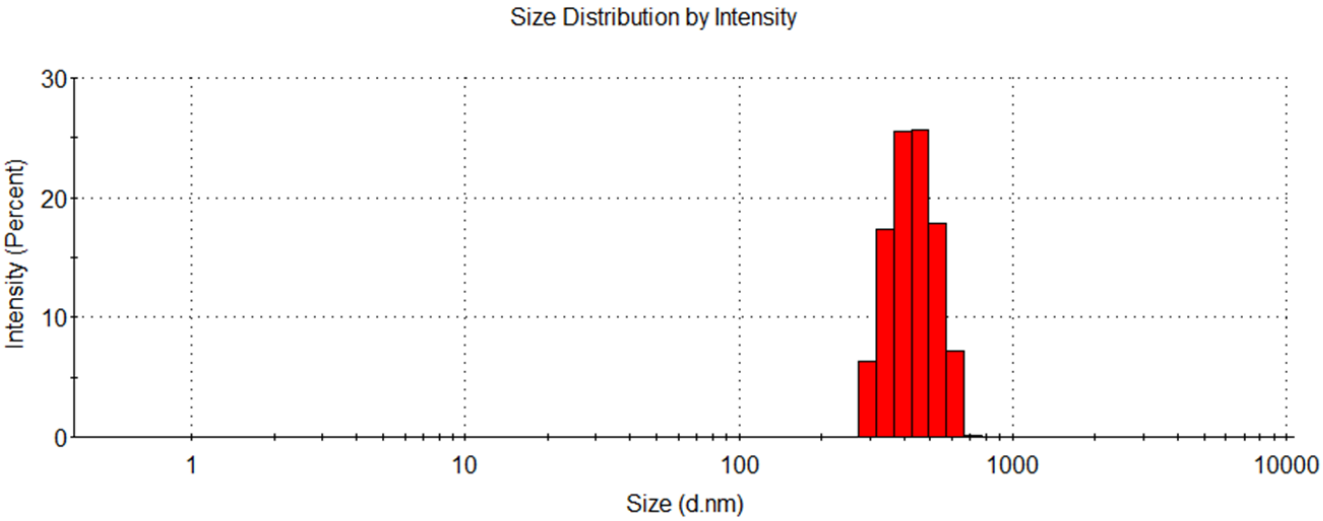

**Figure S5**. Hydrodynamic diameter distribution histograms of **1**(1-2) a) without the genetic cargo (w/o) and b) with the genetic cargo (w/)

a)

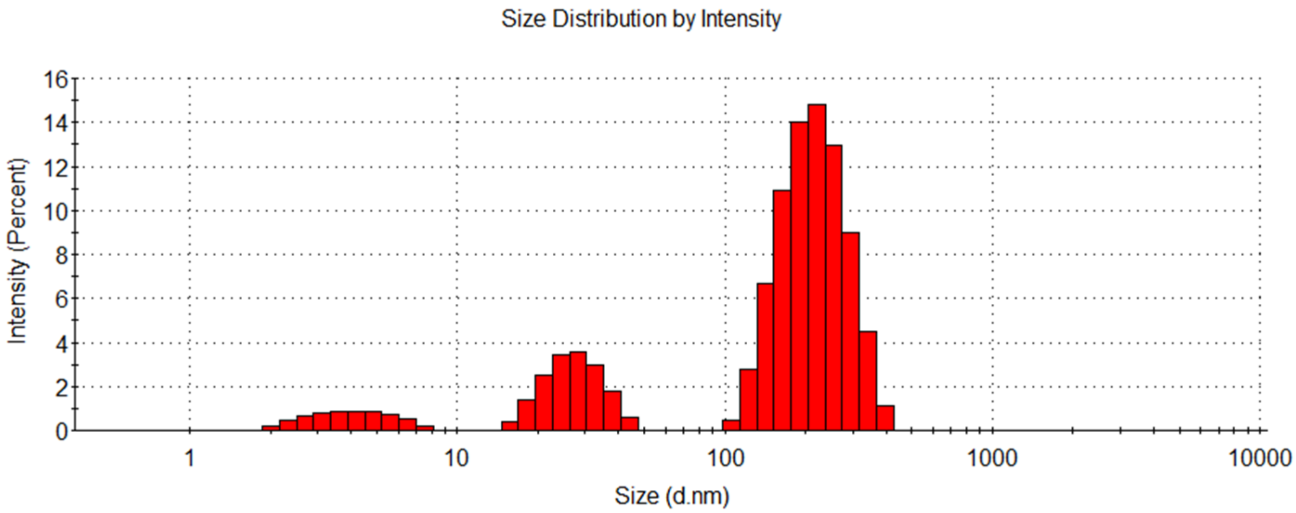


b)

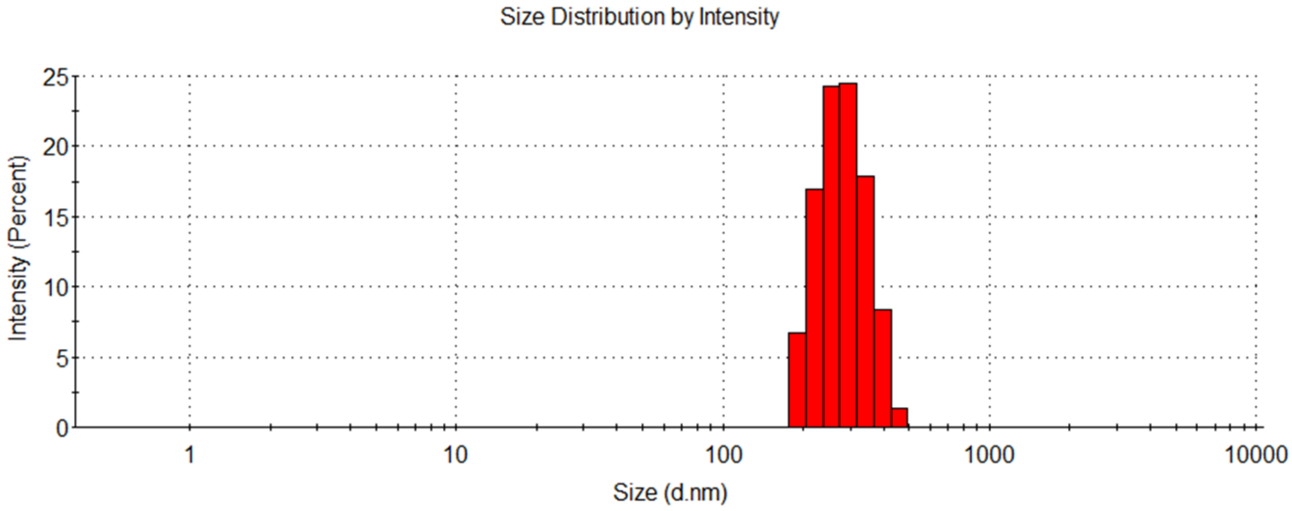

**Figure S6**. Hydrodynamic diameter distribution histograms of **1**(1-4) a) without the genetic cargo (w/o) and b) with the genetic cargo (w/)

a)

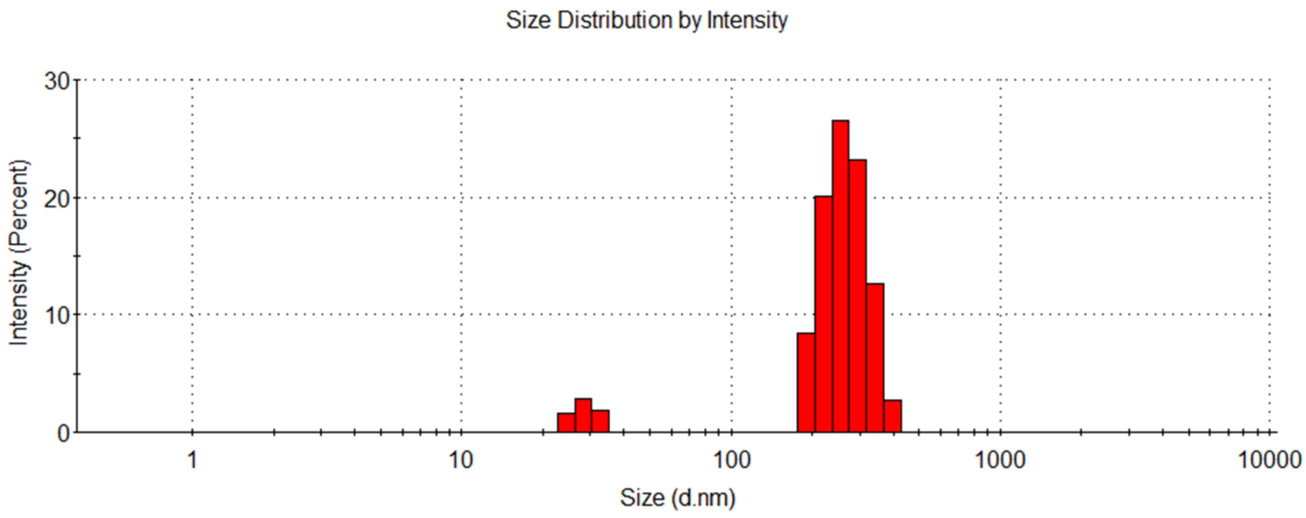


b)

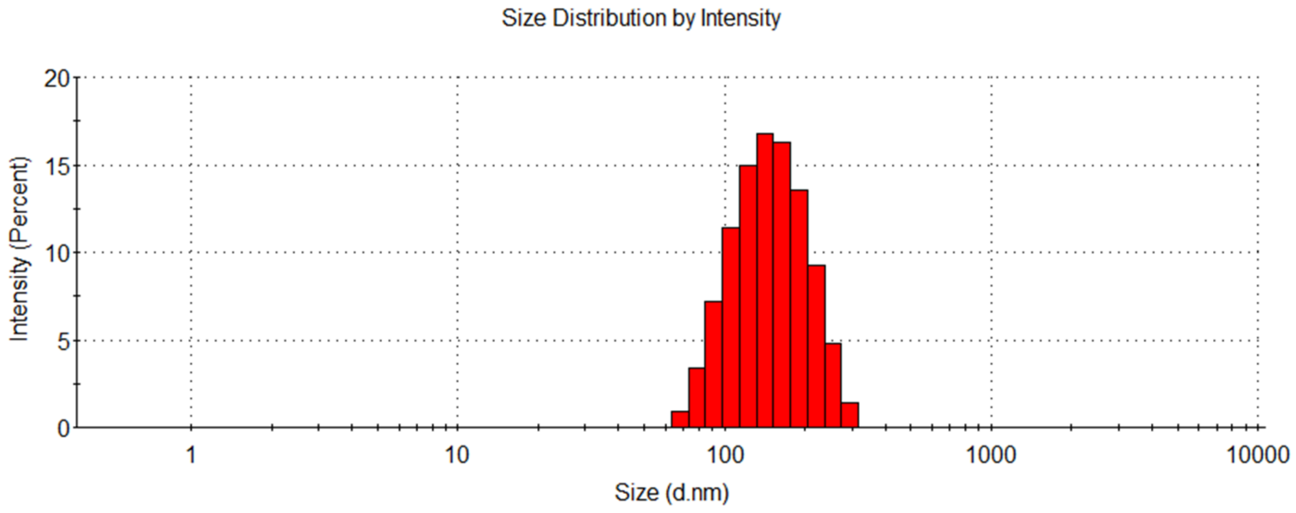

Supplement: Supplementary file 1 [file bm5c00914_si_001.pdf]
